# Loss of Mptx2 alters bacteria composition and intestinal homeostasis potentially by impairing autophagy

**DOI:** 10.1038/s42003-024-05785-7

**Published:** 2024-01-13

**Authors:** Weihui Yan, Shanshan Chen, Ying Wang, Yaying You, Ying Lu, Weipeng Wang, Bo Wu, Jun Du, Shicheng Peng, Wei Cai, Yongtao Xiao

**Affiliations:** 1 Division of Pediatric Gastroenterology and Nutrition, Xinhua Hospital, School of Medicine, Shanghai Jiao Tong University, Shanghai, China; 2 Department of Pediatric Surgery, Xin Hua Hospital, School of Medicine, Shanghai Jiao Tong University, Shanghai, China; 3 Shanghai Key Laboratory of Pediatric Gastroenterology and Nutrition, Shanghai, China; 4 Shanghai Institute of Pediatric Research, Shanghai, China

**Keywords:** Dysbiosis, Bacterial pathogenesis

## Abstract

A recent single-cell survey of the small-intestinal epithelium suggests that mucosal pentraxin 2 (*Mptx2*) is a new Paneth cell marker, but its function and involved mechanism in the Paneth cell are still unknown. Therefore, we create *Mptx2* knockout (*Mptx2*^*−/−*^) mice to investigate its precise effects on intestinal homeostasis using models of lipopolysaccharide (LPS), methicillin-resistant *Staphylococcus aureus* (*MRSA*) peritoneal infection, and dextran sulfate sodium (DSS)–induced intestinal injury and inflammation. We here find that *Mptx2* is selectively expressed in Paneth cells in the small intestines of mice. *Mptx2*^*−/−*^ mice have increased susceptibility to intestinal inflammation and injured. Mptx2 deficiency reduces Paneth cell count and expression of antimicrobial factors, leading to altered intestinal bacteria composition. Loss of *Mptx2* aggravates MRSA infection–induced damage in the intestine while decreasing autophagy in Paneth cells. *Mptx2*^*−/−*^ mice are more vulnerable to LPS-induced intestinal possibly due to inhibition of the autophagy/endoplasmic reticulum (ER) stress pathway. *Mptx2*^*−/−*^ mice are susceptible to DSS-induced colitis that could be ameliorated by treatment with gentamicin or vancomycin antibiotics. In conclusion, Mptx2 is essential to maintain intestinal homeostasis potentially via regulation of autophagy in Paneth cells.

## Abstract (web summary)

Mucosal pentraxin 2 (Mptx2) is identified as a marker of Paneth cells, and its deficiency is shown to trigger microbiota dysbiosis and increased epithelial invasion by bacteria while also reducing secretory autophagy.

## Introduction

Mucosal pentraxin 2 (*Mptx2*) has been proposed to be a member of the pentraxin family due to the high homology of its sequences ( ~ 83% identity in amino acid sequences) to C-reactive protein (*CRP*) and serum amyloid P component protein (*SAP*) in other family members^1^. *CRP* and *SAP* are involved in defending against pathogenic bacteria^2–4^. *Mptx2* is strongly regulated by dietary heme and calcium^5,6^. Recently, a single-cell ribonucleic acid (RNA) sequencing of small-intestinal epithelium indicates *Mptx2* is a novel Paneth cell marker^7^, but its function is still unknown.

Paneth cells are specialized intestinal epithelial cells (IECs) that reside at the bases of small-intestinal crypts and are important maintainers of intestinal homeostasis^8–10^. Impaired autophagy in Paneth cells alters the expression and secretion of antimicrobial factors and intestinal immune responses^11–13^. Therefore, understanding regulatory factors in Paneth cell function is critical to the design of new therapeutic approaches to diseases featuring mucosal inflammation. Autophagy is the process to degrade intracellular entities, such as damaged mitochondria, nuclear fragments, viruses, and bacteria^14^. In the intestinal tract, autophagy is essential to engulf and degrade the invading bacteria, thereby being importantly involved in regulating the intestinal immune response^15^. Indeed, the autophagy-associated genes, including the nucleotide-binding oligomerization domain containing 2 (*NOD2*), and autophagy-related 16 like 1 (*ATG16L1*), have been linked to pathogenesis of inflammatory bowel disease (IBD)^16,17^.

In the current study, to reveal the roles and mechanisms of *Mptx2* in intestinal homeostasis, we began by demonstrating that *Mptx2* was specifically expressed in Paneth cells. We then generated *Mptx2* knockout (*Mptx2*^*−/−*^) mice to study the precise effects of this gene in intestinal inflammation and injury, and to test whether autophagy is involved in mechanisms. Our results showed that *Mptx2* deficiency impaired functions of Paneth cells and thus to cause intestinal inflammation and injury.

## Results

### *Mptx2* was specifically expressed in Paneth cells

**Fig. 1:**
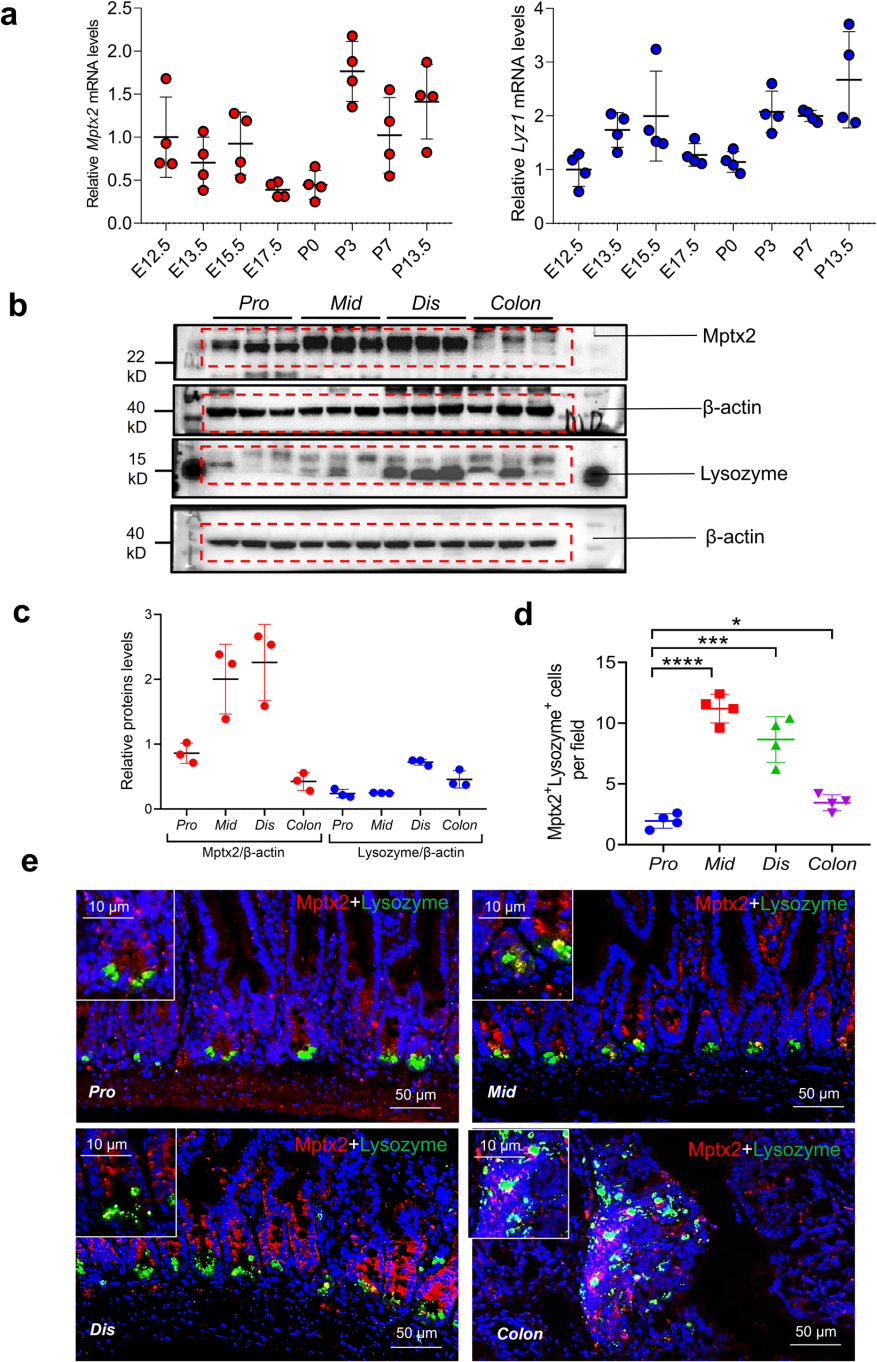
*Mptx2* was selectively expressed in the Paneth cells. **a** Alteration of *Mptx2* mRNA and Lysozyme (*Lyz1*) mRNA from the embryonic stages (E12.5 – 17.5) to the postnatal time (P0 – 13.5) (each group, *n* = 4). **b** Western blot (WB) analysis for Mptx2 and Lysozyme in mouse proximal (pro), middle (mid), distal (dis) small bowel and colon (each group, *n* = 3). Independent experiments at least two times. **c** Quantification of **b**. **d** Quantification of Mptx2 and Lysozyme positive cells in the different segments of mice intestine in **e**. **e** Representative images of immunofluorescence (IF) staining for Mptx2 and Lysozyme in mouse proximal (pro), middle (mid), distal (dis) small bowel and colon (each group, *n* = 4). Unpaired two-tailed Student’s *t* test with or without Welch’s correction analysis for D.*p < 0.05, *** *p* < 0.001,**** *p* < 0.0001.

As shown in Fig. 1a, *Mptx2* messenger RNA (mRNA) was synthesized in the intestines of *Wt* mice from embryonic day 12.5 (E12.5). The levels *Mptx2* mRNA increased significantly starting on postnatal day 0 (P0; Fig. 1a). Similarly, expression of the Paneth cells marker *Lyz1* followed an analogous pattern at the indicated time (Fig. 1a). Under normal conditions, expression of Mptx2 and Lysozyme was higher in the mucosa of mouse middle (mid) and distal (dis) small intestine than in that of proximal (pro) small intestine or colon, but it was not for intestinal stem cell marker Lgr5 (Fig. 1b, c and Supplementary Fig. 1). Consistently, immunofluorescence (IF) staining showed that *Mptx2* protein was exclusively expressed in the intestinal mucosa, especially in those of the mid and dis small intestine (Fig. 1d, e). IF analysis also showed that *Mptx2* protein was mainly co-localized with the Paneth cell marker *Lyz1* in crypt basements (Fig. 1d, e).

### *Mptx2*^*−/−*^ mice were predisposed to intestinal inflammation

**Fig. 2:**
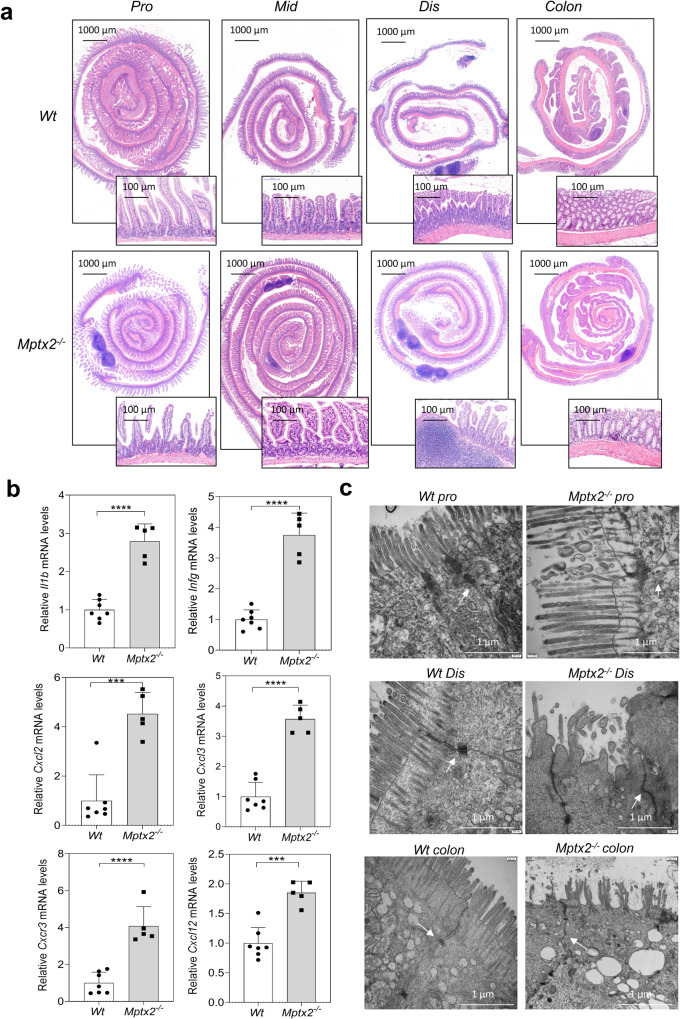
*Mptx2* deficiency triggered intestinal inflammation. **a** Representative images of hematoxylin and eosin (H&E) staining for the proximal (pro), middle (mid), distal (dis) small bowel and colon from both *Mptx2*^*−/−*^ mice (*n* = 12) and *Wt* mice (*n* = 12). **b** qRT-PCR analysis of inflammatory genes mRNA expression in distal (dis) small bowel from both *Mptx2*^*−/−*^ mice (*n* = 5) and *Wt* mice (*n* = 7). **c** Representative images of transmission electron microscopy (TEM) in proximal (pro), distal (dis) small bowel and colon from *Mptx2*^*−/−*^ mice (*n* = 3) and *Wt* mice (*n* = 3). Unpaired two-tailed Student’s *t* test with or without Welch’s correction analysis for **b**.*** *p* < 0.001,**** *p* < 0.0001.

To examine whether *Mptx2* directly affected intestinal homeostasis, we first generated mice lacking the *Mptx2* gene (*Mptx2*^*−/−*^, Supplementary Fig. 2a). Average villus height and crypt depth in *Mptx2*^*−/−*^ mice did not differ from those of their *Wt* littermates (Fig. 2a and Supplementary Fig. 2b). *Mptx2*^*−/−*^ mice had an elevated number of lymphoid structures at the distal part of the small intestines compared to that of *Wt* mice (Fig. 2a and Supplementary Fig. 2c). In agreement with histological findings, inflammatory genes, including *Il1b*, *Ifng*, *Cxcl2*, *Cxcl3*, *Cxcr3*, and *Cxcl12*, were significantly increased in the small-intestinal mucosa of *Mptx2*^*−/−*^ mice compared with their *Wt* littermates (Fig. 2b). Transmission electron microscope (TEM) analysis showed abnormalities in intestinal epithelial intercellular junctions and irregular distribution of microvilli in *Mptx2*^*−/−*^ mice compared with their *Wt* littermates (Fig. 2c).

**Fig. 3:**
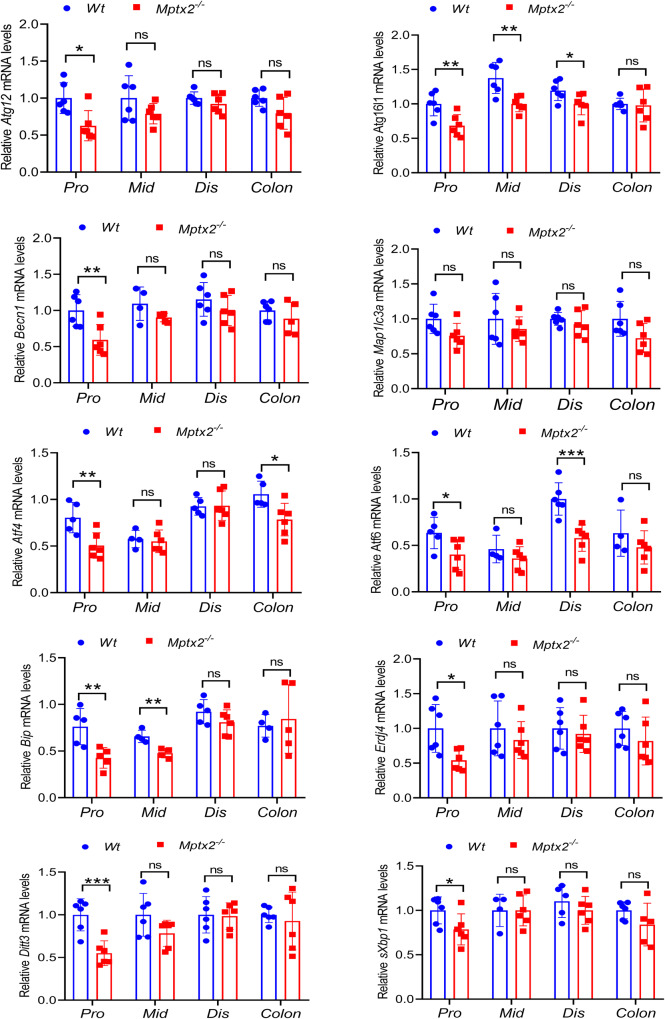
Loss of *Mptx2* impaired the autophagy/endoplasmic reticulum (ER) stress in mice intestine. qRT-PCR analysis of endoplasmic reticulum (ER) stress-autophagy genes mRNA expression in mouse proximal (pro), middle (mid), distal (dis) small bowel and colon from both *Mptx2*^*−/−*^ mice and *Wt* mice (each group, *n* = 4-6). Unpaired two-tailed Student’s *t* test with or without Welch’s correction analysis. ns not significant, *p < 0.05, **p < 0.01, *** *p* < 0.001.

As shown Fig. 3, we observed the autophagy-associated molecules *Atg12*, *Atg16l1*, and *Becn1* mRNA levels significantly decreased in pro small intestines of *Mptx2*^*−/−*^ mice in relation to *Wt* littermates, but the difference of autophagy marker LC3 (*Map1lc3a*) did not arrive at significant level. (Fig. 3). Similarly, ER stress sensor activating transcription factor 4 and 6 (*ATF4*, *ATF6*) mRNA levels especially decreased in the pro small intestines of *Mptx2*^*−/−*^ mice compared with their *Wt* littermates (Fig. 3). Subsequently, downstream markers, including spliced *Xbp1* (*sXbp1*), *Chop* (*Ditt3*), *ERdj4*, and *BiP*, were also reduced evidently in the pro small intestines of *Mptx2*^*−/−*^ mice compared with their *Wt* littermates (Fig. 3). Interestingly, the levels of these mRNAs did not altered evidently in the mid, dis small intestines or in the colon (Fig. 3).

### *Mptx2* deficiency altered intestinal bacteria composition in mice

**Fig. 4:**
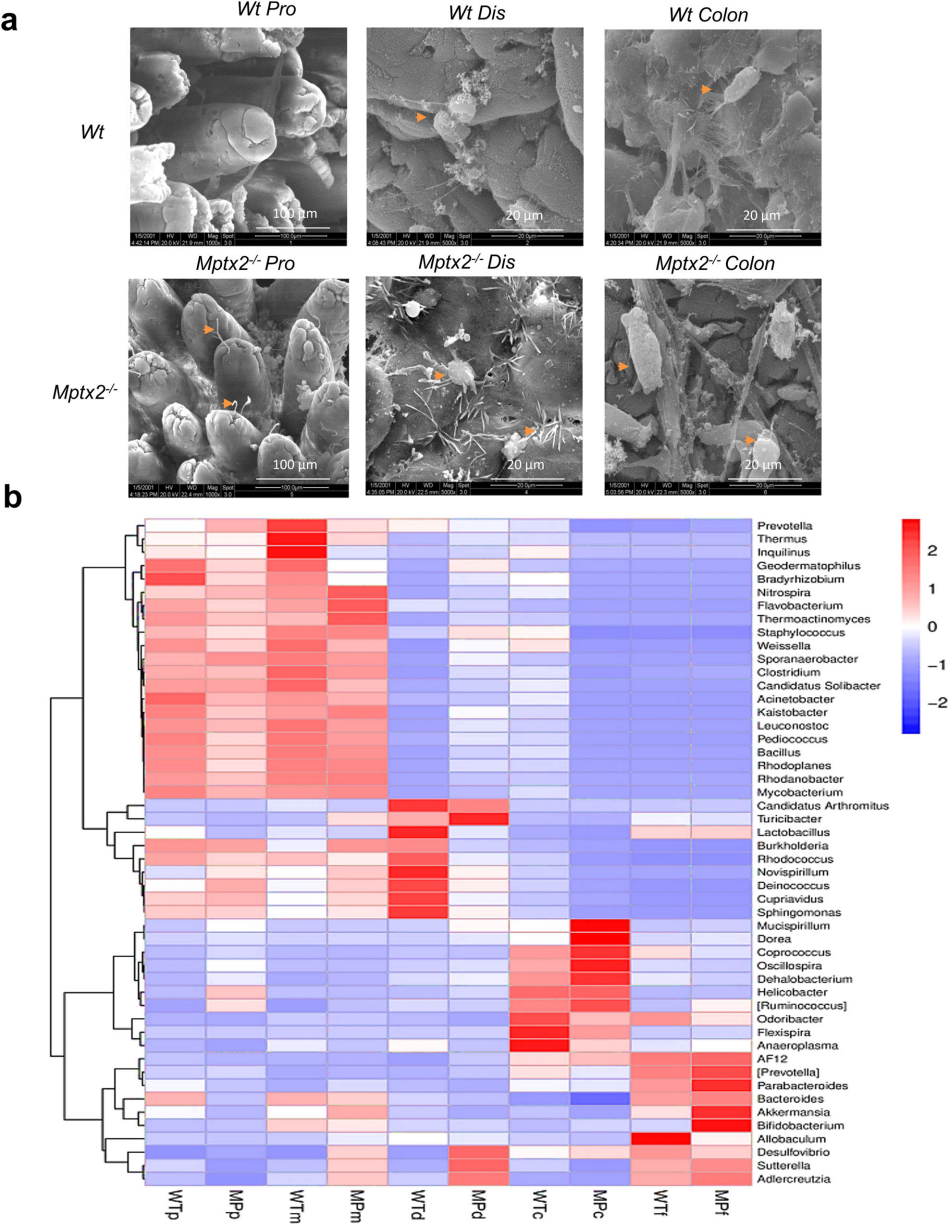
*Mptx2* deficiency altered the intestinal microbiota composition. **a** Representative images of scanning electron microscopy (SEM) analysis for the proximal (pro), distal (dis) small bowel, and colon from both *Mptx2*^*−/−*^ mice (*n* = 3) and *Wt* mice (*n* = 3). **b** The relative abundance of the top bacteria (genus) in the intestinal mucosa and feces of *Mptx2*^*−/−*^ mice and *Wt* mice. WTp: *Wt* mice proximal intestine; MPp: *Mptx2* KO mice proximal intestine; WTm: *Wt* mice middle intestine; MPm: *Mptx2* KO mice middle intestine; WTd: *Wt* mice distal intestine; MPd: *Mptx2* KO mice distal intestine; WTc: *Wt* mice colon; MPc: *Mptx2* KO mice colon; WTf *Wt* mice feces, MPf *Mptx2* KO mice feces (Each group, *n* = 4–6).

Scanning electron microscopy (SEM) analysis showed that an increased number of invading bacteria attached to and aggregated over the epithelial surface in the small intestines and colons of *Mptx2*^*−/−*^ mice (Fig. 4a). We next used 16S rRNA sequencing analysis to explore intestinal bacteria composition in *Mptx2*^*−/−*^ and *Wt* mice (Supplementary Fig. 3). In comparison with their *Wt* littermates, *Mptx2*^*−/−*^ mice showed greater numbers of operational taxonomic units (OTUs) in the genera *Lactobacillus*, *Bifidobacterium, Akkermansia, Bacteroides*, and *Prevotella* in intestines, but reduced abundances of *Staphylococcus*, *Megasphaera*, *Coprococcus*, and *Pseudomonas* in feces (Fig. 4b). Numbers of OTUs in the genera *Staphylococcus, Bacteroides, Lactobacillus*, and increased in the small-intestinal and colonic mucosa in *Mptx2*^*−/−*^ mice (Fig. 4b). We further isolated DNA from the small-intestinal mucosa, colonic mucosa, and fecal content and then analyzed it for the presence of bacteria using PCR with bacterial-genus–specific primers. It showed that the numbers of several bacterial genera increased in the intestinal-mucosa of *Mptx2*^*−/−*^ mice compared to that of *Wt* mice, particularly *all bacteria*, *Bacteroides*, and *Prevotella* (Supplementary Fig. 4).

### *Mptx2* knockout aggravated *MRSA* infection with impairing autophagy in Paneth cell

**Fig. 5:**
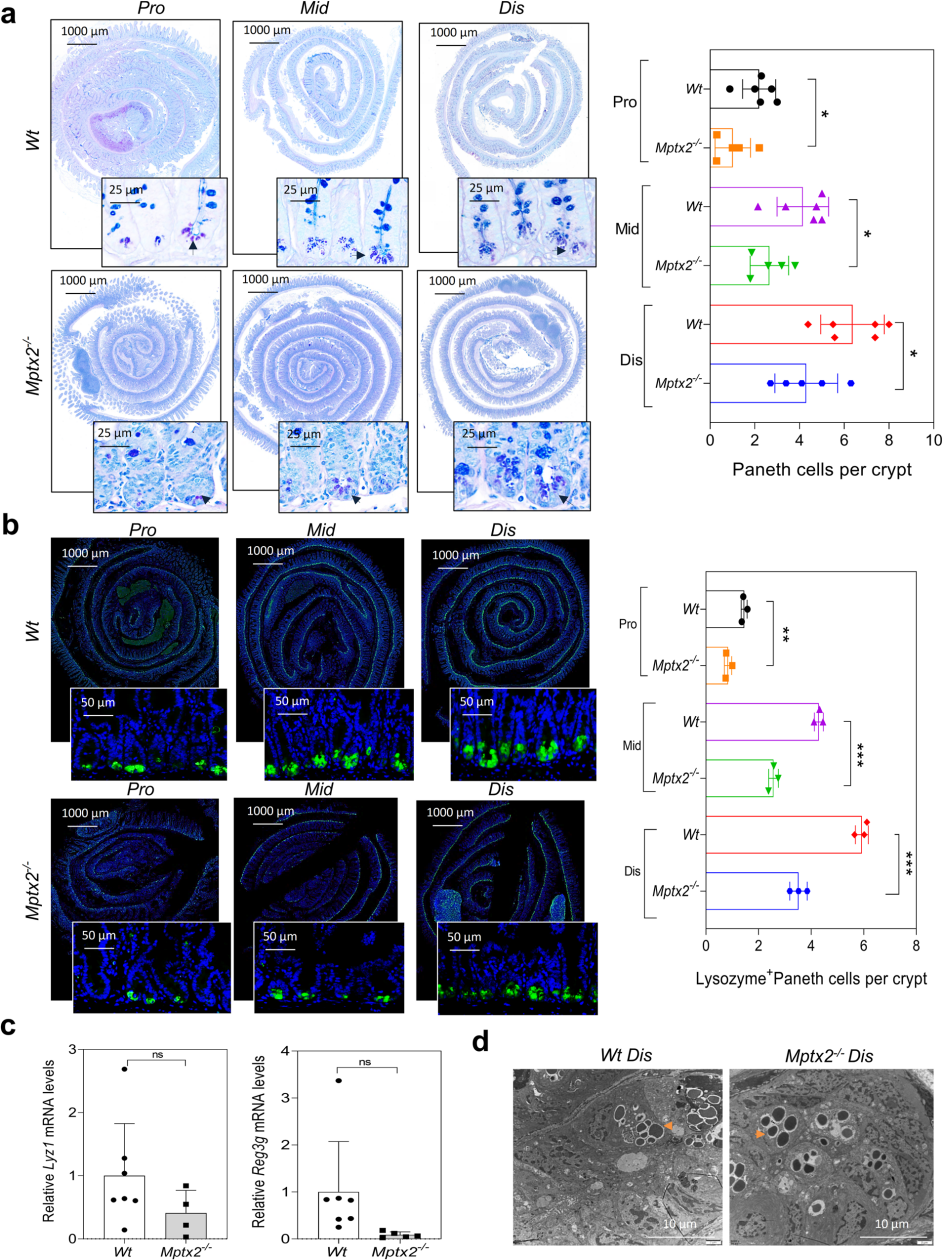
*Mptx2* loss decreased the Paneth cells. **a** Representative images of Alcian blue/periodic acid Schiff base (AB-PAS) staining for the proximal (pro), middle (mid), and distal (dis) small bowel from both *Mptx2*^*−/−*^ mice (*n* = 5) and *Wt* mice (*n* = 6). Quantification of Paneth cells number in both *Mptx2*^*−/−*^ mice (*n* = 5) and *Wt* mice (*n* = 6). **b** Representative images of immunofluorescence (IF) staining of lysozyme for the proximal (pro), middle (mid), and distal (dis) small bowel from both *Mptx2*^*−/−*^ mice (*n* = 3) and *Wt* mice (*n* = 3); Lysosome (green) and DAPI (blue). Quantification of lysozyme-positive cells. **c** Quantitative real-time PCR (qRT-PCR) of *Lyz1* and *Reg3g* in the mucosa of distal (dis) small bowel. **d** Transmission electron microscopy (TEM) analysis for Paneth cells. Arrows indicated granules. Unpaired two-tailed Student’s *t* test with or without Welch’s correction analysis for **a**–**c**. ns not significant, *p < 0.05, **p < 0.01, ***p < 0.001.

Alcian blue-periodic acid Schiff (AB-PAS) staining showed that *Mptx2*^*−/−*^ mice presented Paneth cell loss compared with *Wt* littermates (Fig. 5a). Immunofluorescence (IF) staining-based detection of Lyz1-expressing cells confirmed that Paneth cell count was reduced in the small-intestinal mucosa of *Mptx2*^*−/−*^ mice compared with those of their *Wt* littermates (Fig. 5b). Additionally, quantitative teal-time PCR (qRT-PCR) analysis indicated that representative Paneth cell antimicrobial peptides (AMPs), including *Lyz1* and *Reg3g*, decreased in dis small intestinal mucosa of *Mptx2*^*−/−*^ mice, but it did not reach significant level (Fig. 5c). Representative electron microscope images further showed that *Mptx2*^*−/−*^ mice had more eosinophilic granules (Fig. 5d).

**Fig. 6:**
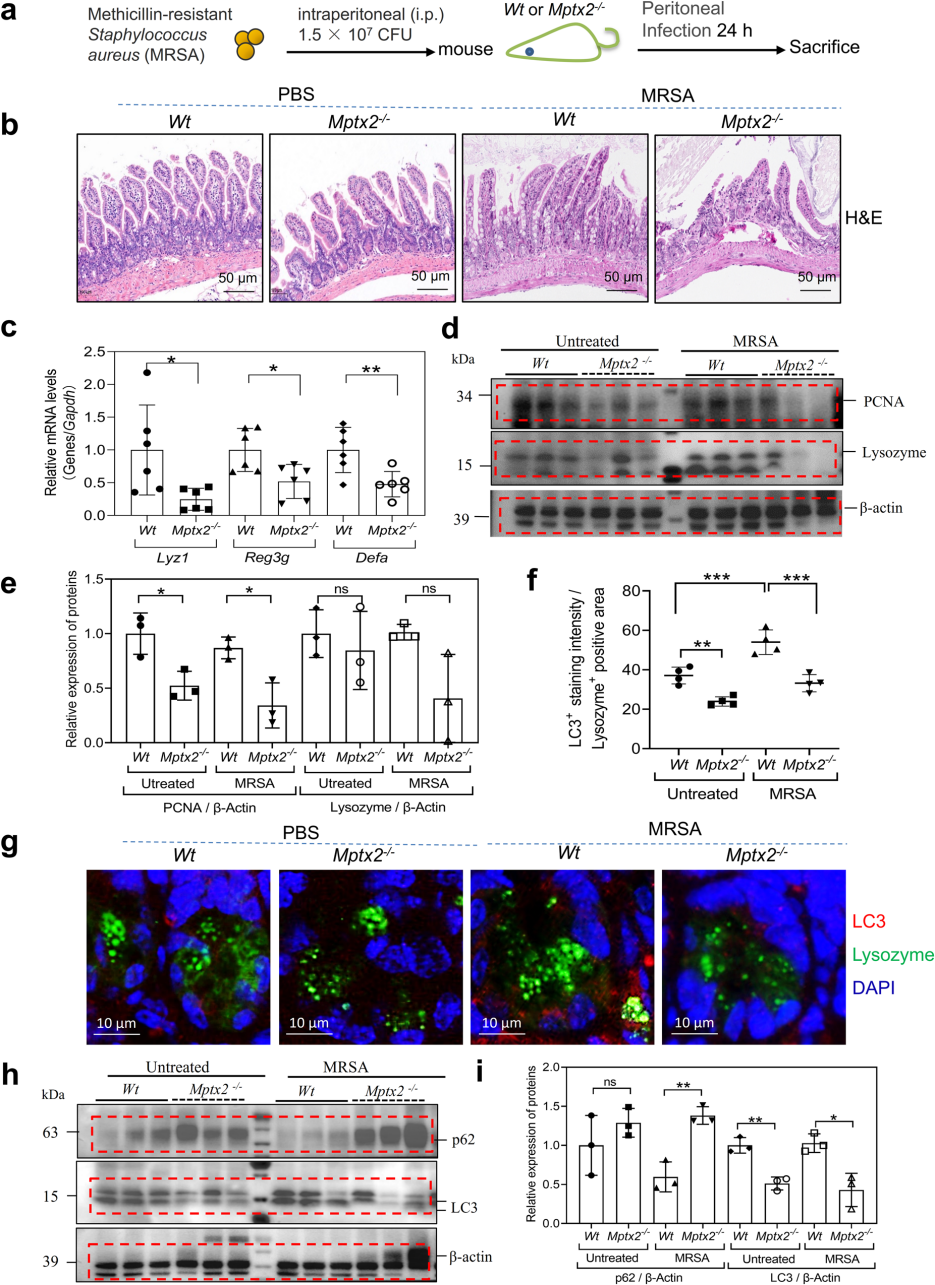
*Mptx2* deficiency worsened the MRSA-infection and disrupted the secretory autophagy in Paneth cells. **a** Schematic of the MRSA-infected mice model. *Mptx2*^*−/−*^ mice (*n* = 7) and *Wt* mice (*n* = 12) mice were infected intraperitoneally with a dose of 1.5 × 10^7^ CFU (colony forming units) of MRSA. This image and every element of this image created by author Dr. X.Y. **b** Representative images of histology in small intestine sections from *Mptx2*^*−/−*^ mice and *Wt* mice (each group, *n* = 4–6). **c** Quantitative real-time PCR (qRT-PCR) of *Lyz1*, *Reg3g*, and defensin alpha (*Defa*) in the mucosa of distal (dis) small bowel followed the MRSA-infection in *Mptx2*^*−/−*^ mice (*n* = 6) and *Wt* mice (*n* = 6). **d** The western blotting (WB) analysis was used to determine the expression levels of PCNA and Lysozyme in the distal mucosa of *Mptx2*^*−/−*^ mice and *Wt* mice. Independent experiments at least two times. **e** Quantification of them against β-actin (each group, *n* = 3). **f**, **g** Representative images of Immunofluorescence analysis for LC3 and Lysosome in the sections of small intestine of *Mptx2*^*−/−*^ mice and *Wt* mice and quantification of them (each group, *n* = 4). **h**, **i** The western blotting (WB) analysis for P62 and LC3 protein in the distal mucosa of *Mptx2*^*−/−*^ mice and *Wt* mice. Quantification of them against β-actin (each group, *n* = 3). These bands from different membranes that have the same protein loading and have their own β-actin as housekeeping protein. Independent experiments at least two times. Unpaired two-tailed Student’s *t* test with or without Welch’s correction analysis for **c**, **e**, **i**. Ordinary One-way ANOVA analysis for F. ns not significant, *p < 0.05, **p < 0.01, ***p < 0.001.

We previously found that administration of recombinant *Mptx2* protein (*rMptx2*) could directly reduce methicillin-resistant *Staphylococcus aureus* (*MRSA*) load in the bloodstream, peritoneal lavage, liver, kidney, spleen, and ileum^18^. In the current study, we showed that *Mptx2*^*−/−*^ mice were more vulnerable to the *MRSA* infection in distal small intestine than *Wt* mice (Fig. 6a, b). Messenger RNA expression of Paneth cell–derived AMPs *Lyz1*, *Reg3g*, and *Defa* was significantly reduced in the distal small intestinal mucosa of *Mptx2*^*−/−*^ mice compared with those of *Wt* mice following *MRSA* infection (Fig. 6c). *Lyz1* and *PCNA* protein levels were also reduced in the distal-small intestinal mucosa of *Mptx2*^*−/−*^ mice *versus* those of *Wt* mice (Fig. 6d, e). *MAP1LC3* (LC3) is an autophagy marker that can capture and eliminate invading bacteria^19^. IF staining indicated that *MRSA* infection increased LC3 puncta in distal-small intestinal mucosa. LC3/Lysozyme colocalization puncta was lower in *Mptx2*^*−/−*^ mice compared to those of *Wt* mice (Fig. 6f, g). Western blot (WB) confirmed that expression of *LC3* was significantly reduced in the distal-small intestinal mucosa of *Mptx2*^*−/−*^ mice compared to those of *Wt* mice (Fig. 6h, i). P62/sequestosome 1 (SQSTM1), a autophagosomal cargo for degradation^20^, increased in distal-small intestinal mucosa of *Mptx2*^*−/−*^ mice (Fig. 6h, i).

### *Mptx2*^*−/−*^ mice increased susceptibility to lipopolysaccharide (LPS)-induced intestinal injury

**Fig. 7:**
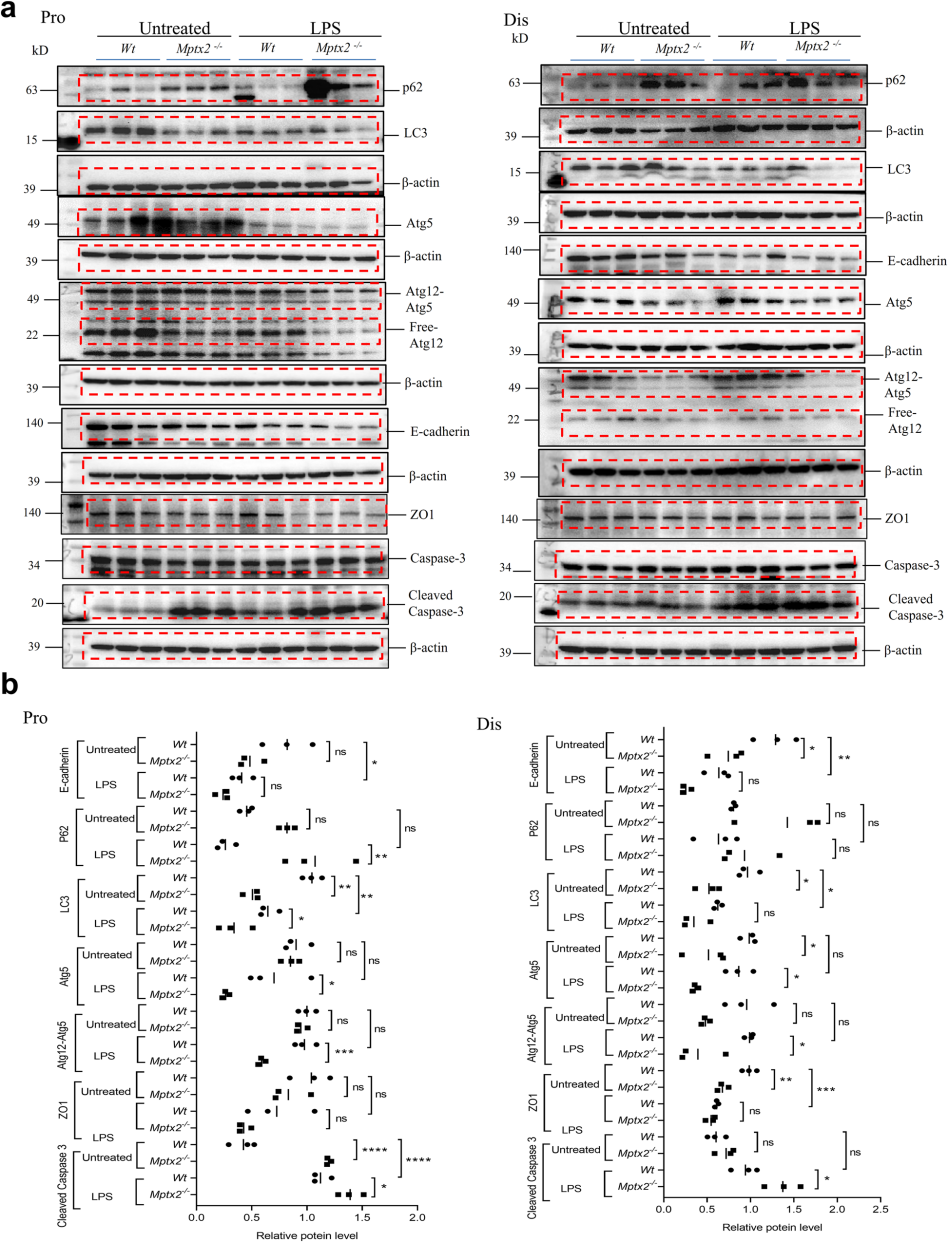
*Mptx2* deficiency increased LPS-induced small intestinal injury with inhibiting the ER stress-autophagy. **a** Representative images of western blotting (WB) analysis for E-cadherin, Atg5, Atg12, LC3,ZO-1,Cleaved-Caspase3 and P62 proteins in small intestines of *Mptx2*^*−/−*^ mice and *Wt* mice with or without LPS treatment. **b** The qualification of WB results in **a**. These bands from different membranes that have the same protein loading and have their own β-actin as housekeeping protein. Independent experiments at least two times. Ordinary One-way ANOVA analysis for B. Aspect ratio of Atg12-Atg5 bands has been adjusted compared to the original image because of limited space. The original images were provided in Supplementary Information. Each group, *n* = 3, ns, not significant, *p < 0.05, **p < 0.01, ***p < 0.001, ****p < 0.0001.

In our LPS-induced mouse sepsis model, *Mptx2* mRNA peaked at 18 h and was gradually silenced by 48 h in the small-intestinal mucosa (Supplementary Fig. 5a). Nineteen hours after LPS injection, we observed more-severe mucosal injury in the small intestines of *Mptx2*^*−/−*^ mice than in those of their *Wt* littermates (Supplementary Fig. 5b, c). In addition, we observed that crypt proliferation was impaired in *Mptx2*^*−/−*^ mice *versus* their *Wt* littermates after LPS administration (Supplementary Fig. 6a, b). Proliferative marker *Yap1*, but not *Lgr5*, decreased in the small-intestinal mucosa of *Mptx2*^*−/−*^ mice (Supplementary Fig. 6c). WB analysis first showed the tight junction proteins E-cadherin and ZO-1 reduced in the pro and dis small intestines of *Mptx2*^*−/−*^ mice compared to their control littermates, but it failed to reach a significant difference (Fig. 7a, b and Supplementary Fig. 7). The apoptotic marker cleaved caspase-3 increased in the pro and dis small intestines of *Mptx2*^*−/−*^ mice compared to their littermates (Fig. 7a, b). It also showed that loss of *Mptx2* impaired the process of the autophagy featured with *Atg5*, *Atg12-Atg5*, and *LC3* proteins were reduced in the pro and dis small intestinal mucosa of *Mptx2*^*−/−*^ mice compared to those of *Wt* mice after LPS-treatment, but the *p62/ SQSTM1* increased in that of *Mptx2*^*−/−*^ mice (Fig. 7a, b).

### Loss of *Mptx2* worsened dextran sulfate sodium (DSS)-induced colitis in mice

**Fig. 8:**
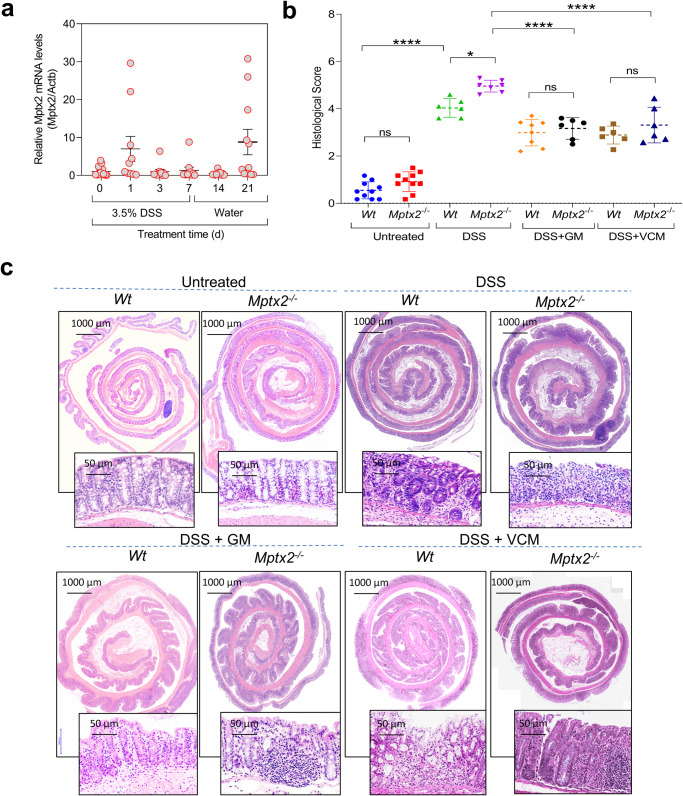
*Mptx2* deficiency exaggerated DSS-induced colitis. **a** qRT-PCR analysis of *Mptx2* mRNA expression in colons of mice (*n* = 10–14) subjected to DSS-induced colitis. **b** Quantification of pathological scores of **c**. **c** Representative images of haematoxylin & eosin (H&E) staining on the colon of mice. Ordinary One-way ANOVA analysis for **b**. each group, *n* = 6–10, ns not significant, *p < 0.05, ****p < 0.0001.

In our DSS-induced colitis and recovery mouse model, high *Mptx2* expression occurred in colonic mucosa during the acute colitis and recovery phases (Fig. 8a). During the process of DSS-induced colitis, *Mptx2*^*−/−*^ mice slightly greater body weight loss than their *Wt* littermates (Supplementary Fig. 8a). The length of colons did not altered evidently between *Mptx2*^*−/−*^ mice and their *Wt* littermates (Supplementary Fig. 8b, c). Histologically, *Mptx2*^*−/−*^ mice had greater colonic-mucosal damage and more inflammatory infiltration than DSS-treated *Wt* mice (Fig. 8b, c). In agreement with histological findings, expression of inflammatory genes, including *Ifng* and *Cxcl2*, was increased in the colonic mucosa of *Mptx2*^*−/−*^ mice after DSS treatment *versus* their DSS-treated *Wt* littermates, but the difference of neither *Tnfa* nor *Cxcl12* arrive at significant level (Supplementary Fig. 9). Moreover, *Mptx2*^*−/−*^ mice had fewer goblet cells in their colons than *Wt* mice in the presence of DSS-treatment (Supplementary Fig. 10). We also found that oral antibiotics (gentamicin, GM or vancomycin, VCM) affected colonic injury and inflammation in *Mptx2*^*−/−*^ mice after DSS treatment (Fig. 8b, c and Supplementary Fig. 10a, b).

## Discussion

To the best of our knowledge, this study confirms *Mptx2* acts as a novel marker of Paneth cells, implying that it might play important roles in intestinal inflammation and homeostasis. We firstly showed that *Mptx2* protein was selectively expressed in Paneth cells in the normal crypt base and that it increased in response to LPS treatment or MRSA infection. *Mptx2* deficiency increased susceptibility to intestinal inflammation and injury might via impairing the autophagy process in Paneth cells.

In the normal intestine, we found that *Mptx2* was exclusively expressed in the mucosa and at higher levels in the small intestine than in the colon. We recently found that *Mptx2* mRNA is also expressed in bone marrow and the spleen^18^. Taken together, these findings suggested that *Mptx2* might be involved in the immune response. In a previous study, a single-cell survey of the small-intestinal epithelium revealed that *Mptx2* might be a new marker for Paneth cells^7^. Indeed, in this study, we confirmed *Mptx2* protein was mainly localized in Paneth cells. Paneth cells are specialized small-intestinal epithelial cells that reside at the bases of crypts and protect the small intestine from enteropathogens by constitutively secreting a broad spectrum of AMPs and bactericidal proteins^8,21^. It is reported that autophagy deficiency within the intestinal leads to an aberrant morphology of Paneth cells^11,12,22^. Our study indicated Mptx2 loss resulted in susceptibility to intestinal inflammation may via the Paneth cells in mice.

*Salmonella* infection has been found to induce expression of AMPs and *Mptx2* in Paneth cells^7^. In the current study, *Mptx2* mRNA increased in the small-intestinal mucosa shortly after LPS treatment. Therefore, *Mptx2* might defend the gut from bacterial infection by modulating Paneth cell. Indeed, we found that loss of *Mptx2* not only reduced Paneth cell count but also inhibited expression of AMPs such as *Lyz1* and *Reg3g*. Subsequently, SEM indicated that *Mptx2*^*−/−*^ mice had greater numbers of invading bacteria that attached to and aggregated over the epithelial surface of the intestine. 16S rRNA sequencing showed that loss of *Mptx2* altered bacteria composition and caused bacteria including the *Staphylococcus*, *Bacteroides*, and *Enterococcus* increased in the intestinal mucosa. Therefore, it is very likely that *Mptx2* defends against invading bacteria via its bactericidal activity and/or by modulating Paneth cell functions. Indeed, we previously found that *Mptx2* exerted bactericidal activity against methicillin-resistant *Staphylococcus aureus* (*MRSA*) both in vitro and in vivo^18^. In the colon, *Mptx2*^*−/−*^ mice aggravated DSS-induced colitis that was ameliorated by GM or VCM treatment, indicating *Mptx2* maintain bacteria homeostasis may via its bactericidal activity. It has been reported that Paneth cells secrete lysozyme to counteract bacterial infection via secretory autophagy^23^. In this study, we found that loss of *Mptx2* aggravated *MRSA* infection with inhibiting the autophagy process in Paneth cells. An impaired ER stress/autophagy crosstalk has been strongly linked to inflammatory bowel disease (IBD)^24–27^. Conditional deletion of intestinal ER stress-marker *Xbp1* leads to a spontaneous enteritis in mice^25^. We observed in the current study that *Mptx2* deficiency exaggerated LPS-induced intestinal injury with reducing *Xbp1* expression and autophagy process. Paneth cells are intercalated between active intestinal stem cells (ISCs) in the small intestine (SI) of mice and humans^28^. Other studies suggest that Paneth cells can constitute a niche for intestinal stem cells in crypts and modulate the regeneration of the intestinal epithelium^29,30^. Therefore, Paneth cells might produce *Mptx2* to promote the regeneration of the intestinal epithelium via constituting the niche. Therefore, we suggest that *Mptx2* might maintain intestinal homeostasis in three ways: (1) acting as an AMP to kill invading bacteria directly, (2) regulating the secretory functions of beyond regulating the microbiota via secretion of AMPs, and (3) contributing to the intestinal repaired via regulating the autophagy/ER-stress.

## Conclusions

Our findings revealed that *Mptx2* was a novel marker of Paneth cells. *Mptx2* deficiency triggered microbiota dysbiosis and increased epithelial invasion by bacteria, leading to greater susceptibility to intestinal inflammation with reducing secretory autophagy. In addition, Paneth cells, which can produce *Mptx2*, contributed to the regeneration of the intestinal epithelium. These findings suggested that *Mptx2* was essential to the functions of Paneth cells and maintained intestinal homeostasis.

## Materials and methods

### Generation of *Mptx2* knockout mice

*Mptx2*^*−/−*^ mice (Δexon 1–2) were generated as in our previous study^18^ via genome engineering mediated by clustered regularly interspaced short palindromic repeats (CRISPRs) and CRISPR-associated protein 9 (Cas9) in C57BL/6 J mice. All procedures involving mice were approved by the Institutional Animal Care and Use Committee of Xinhua Hospital School of Medicine, Shanghai Jiao Tong University (Shanghai, China; No. XHEC-C-F-2022-010). We have complied with all relevant ethical regulations for animal use.

### Production of recombinant *Mptx2* protein

We produced recombinant *Mptx2* protein via the methods described in our previous study^18^. Briefly, the coding sequence of *Mptx2* (NM_001205011) was cloned into the pET-28 vector with an N-terminal 6-histone (6-His) tag (Genechem Co., Ltd, Shanghai, China). We induced *Mptx2* protein via supplementation of 1 mM isopropyl-β-d-thiogalactopyranoside (IPTG) in BL21 (DE3)–competent cells.

### Generation of antibody against *Mptx2*

The protein was purified using a nickel–nitrilotriacetic acid (Ni-NTA) column, a PD MidiTrap G-25 column (GE Healthcare, Chicago, IL, USA), and a Vivaspin 20 centrifugal concentrator (GE Healthcare) according to a published protocol^18^. The purified protein was assessed using Coomassie Brilliant Blue staining. We purified the antibody against *Mptx2* via antigen immunoaffinity. Reactivity was assessed using an enzyme-linked immunosorbent assay (ELISA).

### Dextran sulfate sodium–induced colitis

We used 6-week-old *Mptx2*^*−/−*^ mice (female, *n* = 6; male, *n* = 10) and their wild-type (*Wt*) (female, *n* = 9; male, *n* = 6) littermates for dextran sulfate sodium (DSS)–induced colitis experiments. *Mptx2*^*−/−*^ (female, *n* = 8; male, *n* = 7) mice and *Wt* (female, *n* = 8; male, *n* = 8) mice were untreated as controls. Acute colitis was induced by administration of 2% DSS (36–50 kDa; MP Biomedicals, Solon, OH, USA) in drinking water for 7 days. We monitored changes in mouse body weight (BW) daily. To construct the DSS-induced colitis and recovery mouse model, C57BL/6 mice were induced by 3.5% DSS for 7 days and allowed to recover for 2 weeks (day 0, female, *n* = 8; male, *n* = 7), (day 1, female, *n* = 5; male, *n* = 5), (day 3, female, *n* = 5; male, *n* = 5), (day 7, female, *n* = 5; male, *n* = 5), (recovery 1 week, female, *n* = 5; male, *n* = 5), and (recovery 2 week, female, *n* = 6; male, *n* = 5).

### Lipopolysaccharide–induced systemic inflammation

To induce an acute systemic inflammatory response, we injected C57BL/6 mice ~6 weeks old with lipopolysaccharide (LPS) intraperitoneally (i.p.; 5 mg/kg; #G5032; Wuhan Servicebio Technology Co., Ltd., Wuhan, China). Mice were sacrificed at the time points of 0 h (female, *n* = 8; male, *n* = 7), 3 h (female, *n* = 5; male, *n* = 5), 6 h (female, *n* = 5; male, *n* = 5), 18 h (female, *n* = 5; male, *n* = 5), 24 h (female, *n* = 5; male, *n* = 5), and 48 h (female, *n* = 5; male, *n* = 5)after LPS injection. Control mice received normal saline.

### *Methicillin-resistant Staphylococcus aureus* peritoneal infection

We housed *Wt* and *Mptx2*^*−/−*^ mice ~6 weeks old in a specific-pathogen–free (SPF) unit with access to tap water and pelleted food *ad libitum*. The murine-peritonitis model was established according to the previously described protocol^18,31^. In brief, we infected *Wt* (female, *n* = 5; male, *n* = 7) and *Mptx2*^*−/−*^ mice (female, *n* = 4; male, *n* = 3) i.p. with methicillin-resistant *Staphylococcus aureus* (MRSA; 1.5 × 10^7^ CFU). 2 h post-infection, *Wt* mice received a single dose of 10 μg/ml *Mptx2* recombinant protein (female, *n* = 7; male, *n* = 7). Mice were sacrificed 24 h after infection, and tissues were collected for further analysis.

### Antibiotic treatments

We treated mice with gentamicin (GM; 2 g L^*−*1^; Shandong LuKang Pharmaceutical, Jining, China) or vancomycin (VCM; 500 mg L^*−*1^; Eli Lilly Japan, Kobe, Japan) dissolved in autoclaved drinking water with 2% DSS for 7 days. BW was monitored afterward. GM-DSS-treated mice (*Mptx2*^*−/−*^, female, *n* = 9; male, *n* = 5; *Wt*, female, n = 4; male, n = 5); VCM-DSS-treated mice (*Mptx2*^*−/−*^, female, *n* = 6; male, *n* = 6; *Wt*, female, *n* = 6; male, *n* = 6).

### Intestinal characterization

Intestinal tissues were fixed in 4% paraformaldehyde (PFA) for 24 h and sectioned (4 μm) for hematoxylin and eosin (H&E) staining. We determined villus height and crypt depth using National Institutes of Health (NIH) Image software (NIH, Bethesda, MD, USA) with a microscope (Nikon, Tokyo, Japan). Villus height was measured from five well-oriented villi on each slide; five fields were analyzed per section. We counted goblet cells and quantified mucous secretions using Alcian blue/periodic acid–Schiff (AB/PAS) staining. The count of goblet cells in each villus was calculated from 10 well-oriented villi.

### Histological score

We graded histological changes in the intestinal mucosa as previously described^32,33^. Briefly, histological scores were determined blindly based on the sum of epithelial and infiltration scores. Epithelial scores were as follows: 0 = normal; 1 = loss of goblet cells in small areas; 2 = loss of goblet cells in large areas; 3 = loss of crypts in small areas; and 4 = loss of crypts in large areas. Infiltration scores were as follows: 0 = normal; 1= infiltrate around crypt base; 2 = moderate infiltrate reaching the muscularis mucosae; 3 = extensive infiltration reaching the muscularis mucosae; and 4 = infiltration of the submucosa.

### 5-bromo-2′-deoxyuridine (BrdU) assay

We performed a 5-bromo-2′-deoxyuridine (BrdU) assay according to previously described protocols^34,35^. Briefly, mice were injected with BrdU (50 mg/kg; Solarbio, Beijing, China) 18 h after LPS treatment and euthanized by CO_2_ inhalation 1 h afterward. The entire small intestine was removed, flushed with cold saline, fixed with 4% PFA, and embedded in paraffin. Each group mice count is four (*Mptx2*^*−/−*^, female, *n* = 2; male, *n* = 2; *Wt*, female, *n* = 2; male, *n* = 2). We stained tissue sections (4 μm) via immunofluorescence (IF) using anti-BrdU antibody followed by examination under a fluorescence microscope (Leica, Wetzlar, Germany). BrdU^+^ cells per crypt were counted for 10 random fields per mouse and then averaged.

### Transmission electron microscopy

We prepared intestinal tissues for transmission electron microscopy (TEM) examination followed protocols^36,37^. Briefly, tissues from 6-week-old *Mptx2*^*−/−*^ mice and their *Wt* littermates were fixed with 2.5% glutaraldehyde (GLUT) at room temperature (RT). We then washed the tissues, postfixed them with 1% osmium tetroxide in 0.05 mol/L sodium cacodylate buffer (pH 7.4) at 4 °C for 2 h, stained them with saturated uranyl acetate for 3.5 h at RT, dehydrated them in graded alcohol, and embedded them in Eponate 12 resin (Ted Pella, Inc., Redding, CA, USA). Sections were then cut with a diamond knife and stained with a saturated solution of uranyl acetate in 50% ethanol and lead citrate. We examined and photographed the sections under a Philips CM120 transmission electron microscope (Philips Healthcare, Bothell, WA, USA) at 80 kV.

### Scanning electron microscopy

We cut ~5 mm^2^ gut mucosa from *Mptx2*^*−/−*^ and *Wt* littermate mice and fixed them with 2.5% GLUT overnight at 4 °C. The tissues were rinsed, dehydrated in ethyl alcohol, dried with carbon dioxide, coated with gold, and examined under a Hitachi S-4800 field emission scanning electron microscope (SEM; Hitachi, Tokyo, Japan).

### 16S rRNA gene sequencing

We extracted total microbial genomic-DNA samples from gut mucosa and feces using a DNeasy PowerSoil Kit (QIAGEN, Inc., Venlo, the Netherlands) per the manufacturer’s instructions. Polymerase chain reaction (PCR) amplification of the bacterial 16S ribosomal-RNA (rRNA) gene V4–V5 region was performed using the forward primer 515 F (5′-GTGCCAGCMGCCGCGGTAA-3′) and the reverse primer 907 R (5′-CCGTCAATTCMTTTRAGTTT-3′). We incorporated sample-specific 7-bp barcodes into primers for multiplex sequencing. PCR amplicons were purified using Agencourt AMPure Beads (Beckman Coulter, Indianapolis, IN, USA) and quantified using a PicoGreen Double-stranded Deoxyribonucleic Acid (dsDNA) Assay Kit (Invitrogen, Carlsbad, CA, USA). After the individual-quantification step, amplicons were pooled in equal amounts, and paired-end (PE) 2 × 300 bp sequencing was performed on an Illumina MiSeq platform with MiSeq Reagent Kit version 3 (Illumina, Inc., San Diego, CA, USA) at Shanghai Personal Biotechnology Co., Ltd. (Shanghai, China). We used the Quantitative Insights into Microbial Ecology (QIIME; https://qiime.org) version 1.8.0) pipeline to process sequencing data, as previously described^38^. Sequence data were mainly analyzed using QIIME and R software version 3.2.0 (R Foundation for Statistical Computing, Vienna, Austria).

### Quantitative real-time polymerase chain reaction amplification of 16S rRNA genes

After recording weights, we extracted bacterial DNA from colonic content using a QIAamp Fast DNA Mini Kit (QIAGEN). Quantitative real-time PCR (qRT-PCR) was performed on an ABIViiA 7 system (Applied Biosystems [Thermo Fisher Scientific, Waltham, MA, USA]) using an SYBR Green Universal Master Mix Kit (Thermo Fisher). We used the following primers, which were modified from a previous study’s sets^39^: *all bacteria*, F-5′-CGGTGAATACGTTCCCGG-3′ and R-5′-TACGGCTACCTTGTTACGACTT-3′; *Bifidobacterium*, F-5′-CTCCTGGAAACGGGTGG-3′ and R-5′-GGTGTTCTTCCCGATATCTACA-3′; *Lactobacillus*, F-5′-TGGAAACAGRTGCTAATACCG-3′ and R-5′-GTCCATTGTGGAAGATTCCC-3′; *Bacteroides*, F-5′-GAGAGGAAGGTCCCCCAC-3′ and R-5′-CGCTACTTGGCTGGTTCAG-3′; *Prevotella*, F-5′-CACRGTAAACGATGGATGCC-3′ and R-5′-GGTCGGGTTGCAGACC-3′; *Escherichia/Shigella*, F-5′-GAGTAAAGTTAATACCTTTGCTCATTG-3′ and R-5′-GAGACTCAAGCTKRCCAGTATCAG-3′; *Helicobacter*, F-5′-CTATGACGGGTATCCGGCC-3′ and R-5′-TCGCCTTCGCAATGAGTATT-3′; *Staphylococcus*, F-5′-TTTGGGCTACACACGTGCTACAATGGACAA-3′ and R-5′-AACAACTTTATGGGATTTGCWTGA-3′; Commensal segmented filamentous bacteria (Com-SFB), F-5′-AGGAGGAGTCTGCGGCACATTAGC-3′ and R-5′-CGCATCCTTTACGCCCAGTTATTC-3′; and murine SFB (Mus-SFB), F-5′-TGAGCGGAGATATATGGAGC-3’ and R-5′-CATGCAACTATATAGCTATATGCGG-3′.

### Quantitative real-time polymerase chain reaction

Total RNA was extracted from intestinal-mucosal tissues using an RNeasy kit (QIAGEN) per the manufacturer’s protocol. We quantified RNA using a NanoDrop spectrophotometer (Applied Biosystems). A High Capacity Complementary DNA (cDNA) Reverse Transcription Kit (Applied Biosystems) was employed for reverse transcription using 2 μg RNA. Subsequently, we performed real-time PCR reactions using a ViiA 7 Real-Time PCR System with PowerUp SYBR Green Master Mix Kit (both, Applied Biosystems). PCR reactions were incubated in a 384-well plate at 95 °C for 10 min, followed by 40 cycles at 95 °C for 15 s and 60 °C for 1 min. All samples were assayed in triplicate, and data were normalized to endogenous control β-actin. We calculated relative RNA expression levels using the ^ΔΔ^Ct method. Primers, which were modified from previous studies^40–42^ and synthesized by Invitrogen (Shanghai, China), were as follows in Supplementary Table 1.

### Western blotting

For Western blotting (WB), we homogenized ~50 mg tissue in 500 μL radioimmunoprecipitation assay (RIPA) buffer (Invitrogen, Carlsbad, CA, USA) supplemented with a protease inhibitor cocktail (Servicebio). Bicinchoninic acid (BCA) reagent (Pierce Biotechnology [Thermo Fisher]) was used to determine the protein concentration. Next, we separated equal amounts of protein onto 10% NuPAGE Bis-Tris gels (Invitrogen) and transferred them to polyvinylidene difluoride (PVDF) membranes (MilliporeSigma, Burlington, MA, USA). After blocking in 5% nonfat milk, membranes were incubated with primary antibodies overnight at 4 °C. Information for primary antibodies used in this study was showed Supplementary Table 2. We then washed the membranes three times with tris-buffered saline containing 0.1% Polysorbate 20 (TBST) and incubated them with secondary antibodies. After final washes of the tissues with TBST, we detected signals using an Electrochemiluminescence (ECL) Reagent Kit (Pierce). All of original WB bands were provided in Supplementary information.

### Immunofluorescence (IF) assay

Immunofluorescence (IF) assay was performed as we described previously^43^. Briefly, the intestinal tissues from were immediately fixed in 4% paraformaldehyde for 24 h and went through dehydration, clearing and paraffin embedding. Sections were mounted on positively charged slides after cutting at 4 μm thick, were then incubated with xylol and descending concentrations of ethanol. After antigen retrieval, blocking was performed using 5% bovine serum albumin for 30 min at room temperature. The primary antibodies were incubated in a humid chamber for overnight at 4 °C. The following day, the slides were incubated with the secondary antibody for 50 min at room temperature away from light after washing with phosphate-buffered saline (PBS). The information for primary antibodies used is listed in Supplementary Table 2.

### Statistics and reproducibility

Numerical source data for all charts are provided in Methods and Figure legends. Statistical tests were performed using GraphPad Prism 8 Software (GraphPad, San Diego, CA) via two–tailed unpaired t test between two groups and one-way ANOVA for multiple comparisons. Each mouse was assessed as an individual sample. All data were obtained by performing at least 3 independent experiments with representative data shown and expressed as the mean ± standard error of the mean (SEM). *P* values < 0.05 were considered statistically significant. Significance levels were split further as to **P < 0.01, ***P < 0.001, ****P < 0.0001.

### Reporting summary

Further information on research design is available in the Nature Portfolio Reporting Summary linked to this article.

### Supplementary information

Supplementary Information

Description of Additional Supplementary Files

Supplementary data 1

Reporting Summary

## Data Availability

The data generated or analyzed during this study are available from the corresponding author upon reasonable request. Source data, as well as statistical analysis for all graphs, are provided in the Excel file Supplementary Data 1. Source images for representative Western blots shown in figures are provided in Supplementary Figure 11 in Supplementary information. Source data of 16S rRNA gene sequencing is deposited in National Center for Biotechnology Information BioProject (Accession: PRJNA1051373).
