# Supplementary material for: Loss of Mptx2 alters bacteria composition and intestinal homeostasis potentially by impairing autophagy
Source: Commun Biol. 2024 Jan 13;7:94. doi: 10.1038/s42003-024-05785-7 (PMC10787791; doi:10.1038/s42003-024-05785-7)

Supplementary information

Loss of Mptx2 alters bacteria composition and intestinal homeostasis potentially by impairing autophagy

Weihui Yan, Shanshan Chen, Ying Wang, Yaying You, Ying Lu, Weipeng Wang, Bo Wu, Jun Du, Shicheng Peng, Wei Cai and Yongtao Xiao

Supplemental Information contents:

Supplementary Tables 1 - 2, Page 2 – Page 4

Supplementary Figures 1 -10, Page 5 – Page 18

Supplementary Figure 11, Page 19 – Page 34

1. Supplementary Tables

Supplementary Table 1 Sequences of primers

Atf6	Forward (5'-3')	GCGGATGATAAAGAACCGAGAG
	Reverse (5'-3')	ACAGACAGCTCTTCGCTTTG
Bip	Forward (5'-3')	TCATCGGACGCACTTGGAA
	Reverse (5'-3')	CAACCACCTTGAATGGCAAGA
Ditt3	Forward (5'-3')	GTCCCTAGCTTGGCTGACAGA
	Reverse (5'-3')	TGGAGAGCGAGGGCTTTG
Erdj4	Forward (5'-3')	GGCGCACAGGTTATTAGAAATG
	Reverse (5'-3')	TCGCTCTGAGGCAGACTTTG
sXbp1	Forward (5'-3')	AAGAACACGCTTGGGAATGG
	Reverse (5'-3')	CTGCACCTGCTGCGGAC
uXbp1	Forward (5'-3')	TGGCCGGGTCTGCTGAGTCCG
	Reverse (5'-3')	GTCCATGGGAAGATGTTCTGG
Gapdh	Forward (5'-3')	CCACTCACGGCAAATTCAAC
	Reverse (5'-3')	CTCCACGACATACTCAGCAC
Atg12	Forward (5'-3')	CACACATGGCAGCACTCCTA
	Reverse (5'-3')	TTCCCCCAGAGGTGAGACAA
Atg16l1	Forward (5'-3')	ATTCTTCTGATGCTGCCAGGAG
	Reverse (5'-3')	CGCATCGAAGACATACGAGG
Atf4	Forward (5'-3')	GGGTTCTGTCTTCCACTCCA
	Reverse (5'-3')	AAGCAGCAGAGTCAGGCTTTC
Mptx2	Forward (5'-3')	GCTCTATGTTGGGAATTCGGGA
	Reverse (5'-3')	CAATCCCAGAGCCAGACTCC
Lyz1	Forward (5'-3')	GGAATGGATGGCTACCGTGG
	Reverse (5'-3')	CATGCCACCCATGCTCGAAT
Defa	Forward (5'-3')	GTCGCTGAACATGGAGACCA
	Reverse (5'-3')	GTCATCAGGCACCAGCATCA
Reg3g	Forward (5'-3')	CAGACAAGATGCTTCCCCGT
	Reverse (5'-3')	GCAACTTCACCTTGCACCTG
Ifng	Forward (5'-3')	TCAAGTGGCATAGATGTGGAAGAA
	Reverse (5'-3')	TGGCTCTGCAGGATTTTCATG
Cxcl2	Forward (5'-3')	GCTGTCCCTCAACGGAAGAA
	Reverse (5'-3')	CAGGTACGATCCAGGCTTCC
Cxcl3	Forward (5'-3')	GAAAGGAGGAAGCCCCTCAC
	Reverse (5'-3')	ACACATCCAGACACCGTTGG
Cxcr3	Forward (5'-3')	ATCAGCGCTTCAATGCCAC
	Reverse (5'-3')	TGGCTTTCTCGACCACAGTT
Cxcl12	Forward (5'-3')	GGTGCTCAAACCTGACGGTA
	Reverse (5'-3')	GGCAGCTCCTCTTTGGCTTA
Tnfa	Forward (5'-3')	TCCAGGCGGTGCCTATGT

	Reverse (5'-3')	CACCCCGAAGTTCAGTAGACAGA
Il10	Forward (5'-3')	AGGCGCTGTCATCGATTTCT
	Reverse (5'-3')	ATGGCCTTGTAGACACCTTGG
Tgfb1	Forward (5'-3')	AGGGCTACCATGCCAACTTC
	Reverse (5'-3')	CCACGTAGTAGACGATGGGC
Lypd8	Forward (5'-3')	GCCTTCACTGTCCATCTATTT
	Reverse (5'-3')	GTGACCATAGCAAGACATGCA
Muc2	Forward (5'-3')	CAGTTTATTCCTGTGTGCCCAAGG
	Reverse (5'-3')	GGCTTCAGAATAATGTACTGCTGC
Yap1	Forward (5'-3')	TTCGGCAGGCAATACGGAAT
	Reverse (5'-3')	TGCTCCAGTGTAGGCAACTG
Lgr5	Forward (5'-3')	CATCACACTGTCACTGTGAGC
	Reverse (5'-3')	GGTAGCTGACTGATGTTGTTC
β-actin	Forward (5'-3')	CACTGTCGAGTCGCGTCC
	Reverse (5'-3')	CGCAGCGATATCGTCATCCA
Map1lc3a	Forward (5'-3')	TTCGGGTTGCTCTTTTGGGT
	Reverse (5'-3')	GACAGGCAAGGGCCTAACAA

Supplementary Table 2 Antibody information

Antibody	Source	Catalog#	Application/dilution
β-actin	Cell Signaling Technology ,CST	#4970S	WB (1:2000)
Mptx2	This study	N/A	IF (1:100); WB (1:1000)
p62/SQSTM1	R&D Systems	#MAB8028	WB (1 µg/mL)
Microtubule-associated protein 1, light chain 3/LC3	Cell Signaling Technology ,CST	#12741S	WB (1:1000); IF (1:200)
Proliferating cell nuclear antigen, PCNA	Bioss	#bsm-33035M	WB (1:1000)
LGR5	Abcam	#ab75732	WB (1:1000)
Lysozyme	Servicebio	#GB11345	WB (1:1000); IF (1:100)
E-cadherin	Cell Signaling Technology ,CST	#3195P	WB (1:1000)
ATG12	Cell Signaling Technology ,CST	#4180P	WB (1:1000)
ZO-1	Bioss	#bs-1329R	WB (1:1000)
ZO-1	Servicebio	#GB11195	IHC (1:500)
Cleaved-Caspase3	Cell Signaling Technology ,CST	9664P	WB (1:1000)
Caspase3	Cell Signaling Technology ,CST	9665S	WB (1:1000)
ATG5	Cell Signaling Technology ,CST	#12994s	WB (1:1000)

2. Supplementary Figures

Supplementary Figure 1 Expression of Mptx2 mRNA and Lgr5 protein in the mice intestine. a Quantification of *Mptx2* mRNA in the mucosa of mouse proximal (pro), middle (mid), distal (dis) small bowel and colon (each group, n = 6). **b** Representative images of the DNA agarose gels for *Mptx2* mRNA quantification. **c** Western-blot analysis for Lgr5 in mouse proximal (pro), middle (mid), distal (dis) small bowel and colon (each group, n = 3). Independent experiments at least two times. **d** Quantification of panel (**c**).

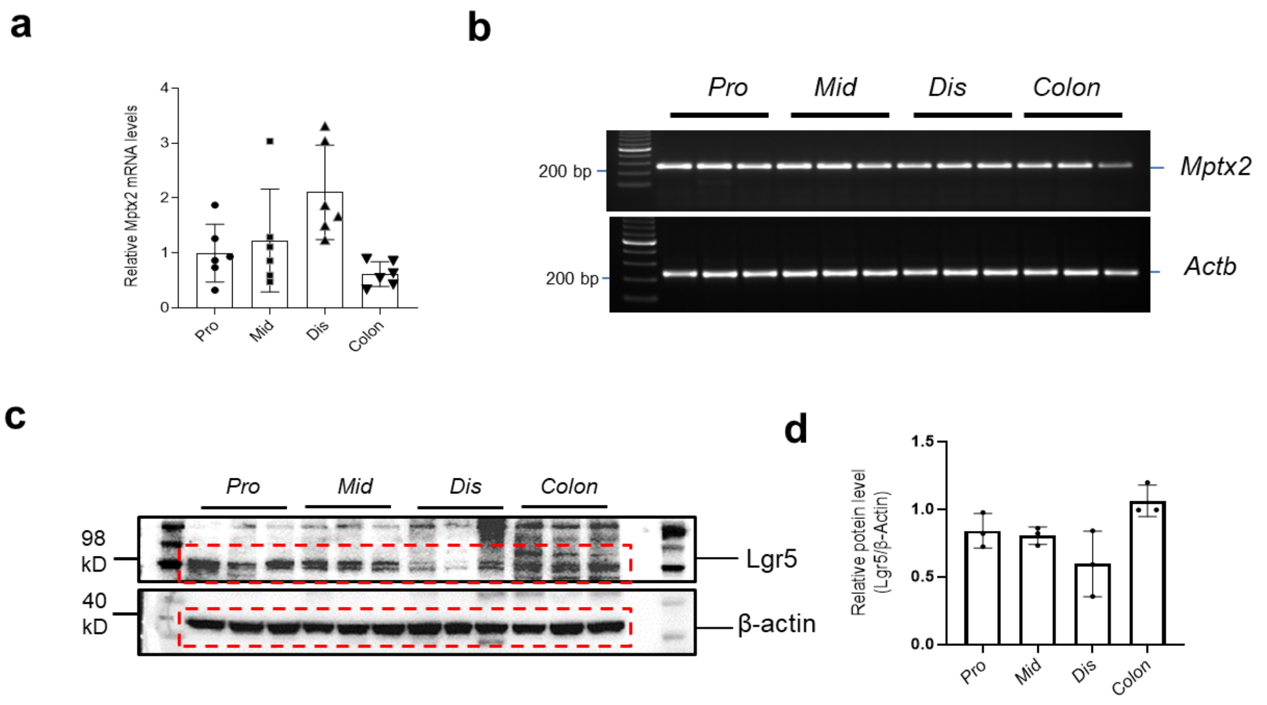

Supplementary Figure 2 The quantification of intestinal villus height, crypt depth, and lymphoid count in the *Wt* and *Mptx2*$^{-/-}$ mice. a Representative images of western blotting (WB) analysis for Mptx2 and β-actin in dis small intestines of *Mptx2*$^{-/-}$ mice and *Wt* mice with or without LPS treatment. (Each group, n = 3). **b** The quantification of intestinal villus height and crypt depth in the *Wt* and *Mptx2*$^{-/-}$ mice (Each group, n = 12 - 16). **c** The quantification of intestinal lymphoid count in the *Wt* and *Mptx2*$^{-/-}$ mice (Each group, n = 5 - 8). Unpaired two-tailed Student's t test with or without Welch's correction analysis for panel (**b**) and (**c**). ns, not significant, ** $p<0.01$.

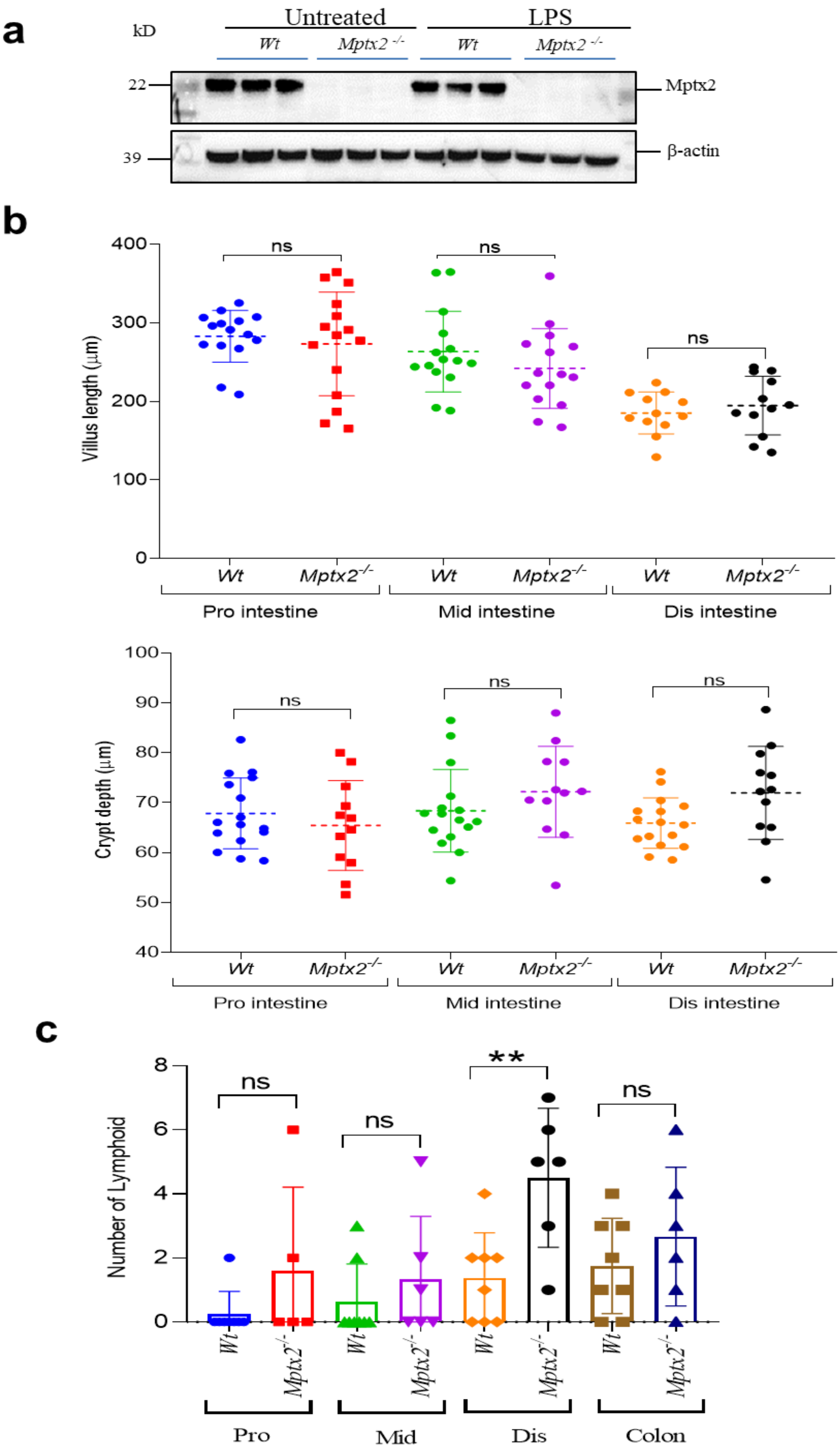
a
kD
Untreated
LPS
Wt
Mptx2 -/-
Wt
Mptx2 -/-
22
39
Mptx2
β-actin
b
Villus length (μm)
400
300
200
100
0
ns
ns
ns
Wt
Mptx2-/-
Wt
Mptx2-/-
Wt
Mptx2-/-
Pro intestine
Mid intestine
Dis intestine
Crypt depth (μm)
100
90
80
70
60
50
40
ns
ns
ns
Wt
Mptx2-/-
Wt
Mptx2-/-
Wt
Mptx2-/-
Pro intestine
Mid intestine
Dis intestine
c
Number of Lymphoid
8
6
4
2
0
ns
ns
**
ns
Wt
Mptx2-/-
Wt
Mptx2-/-
Wt
Mptx2-/-
Wt
Mptx2-/-
Pro
Mid
Dis
Colon

Supplementary Figure 3 The abundance and diversity of microbiota in the *Wt* and *Mptx2*$^{-/-}$ mice. WTp: *Wt* mice proximal intestine; MPp: *Mptx2* KO mice proximal intestine; WTm: *Wt* mice middle intestine; MPm: *Mptx2* KO mice middle intestine; WTd: *Wt* mice distal intestine; MPd: *Mptx2* KO mice distal intestine; WTc: *Wt* mice colon; MPc: *Mptx2* KO mice colon; WTf: *Wt* mice feces; MPf: *Mptx2* KO mice feces. (Each group, n = 4 - 6)

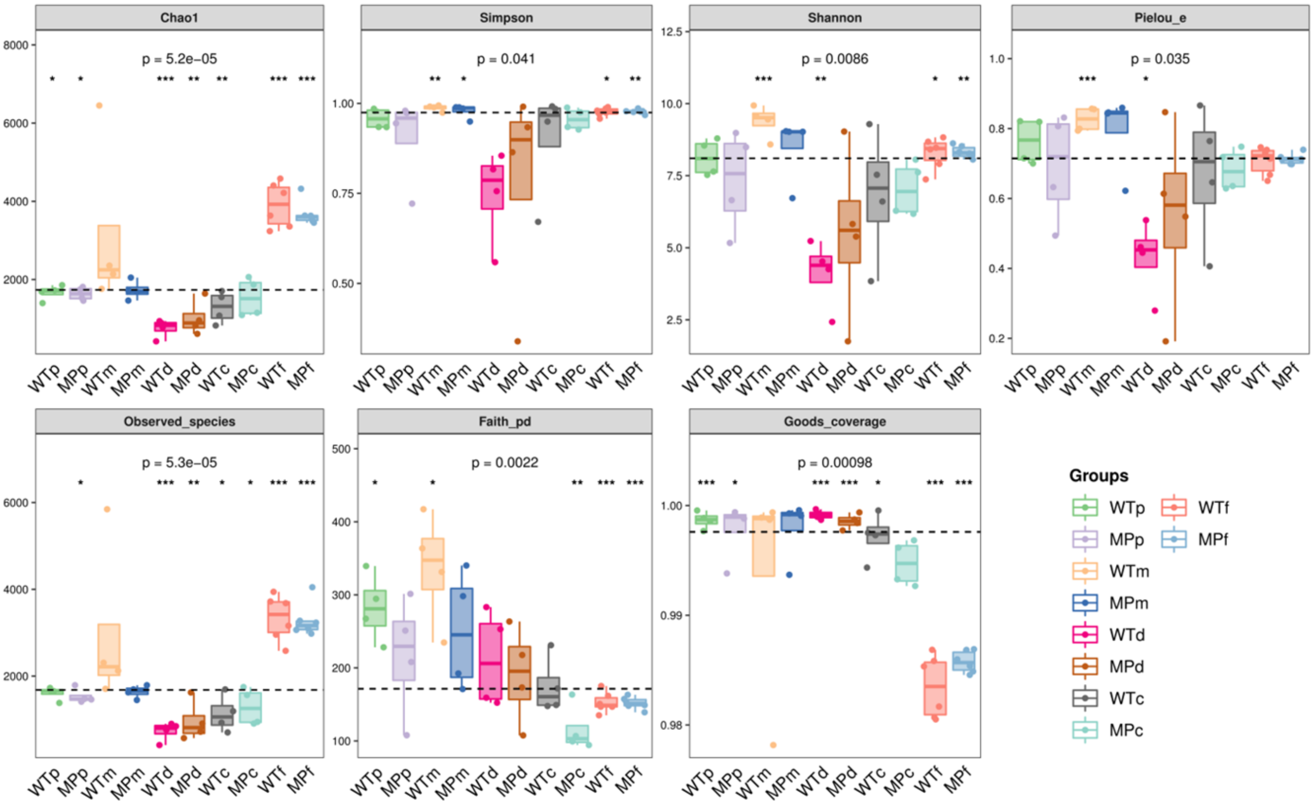

Supplementary Figure 4 Quantitative PCR of bacterial DNA isolated from feces and intestinal mucosa from *Mptx2*$^{-/-}$ mice and *Wt* mice. Com SFB: commensal segmented filamentous bacteria; Mus SFB: mouse segmented filamentous bacteria. (Each group, n =3 - 8).Unpaired two-tailed Student's t test with or without Welch's correction analysis for Supplementary Figure 4 . ns, not significant, * p<0.05.

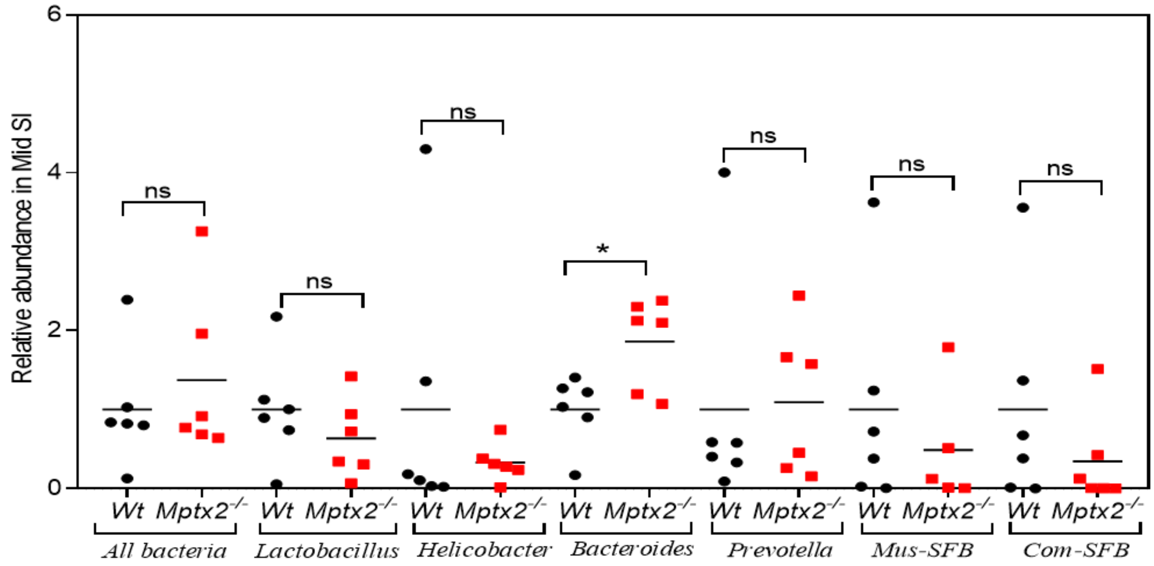
Relative abundance in Mid SI
6
4
2
0
ns
ns
ns
*
ns
ns
ns
Wt Mptx2-/-
All bacteria
Lactobacillus
Helicobacter
Bacteroides
Prevotella
Mus-SFB
Com-SFB

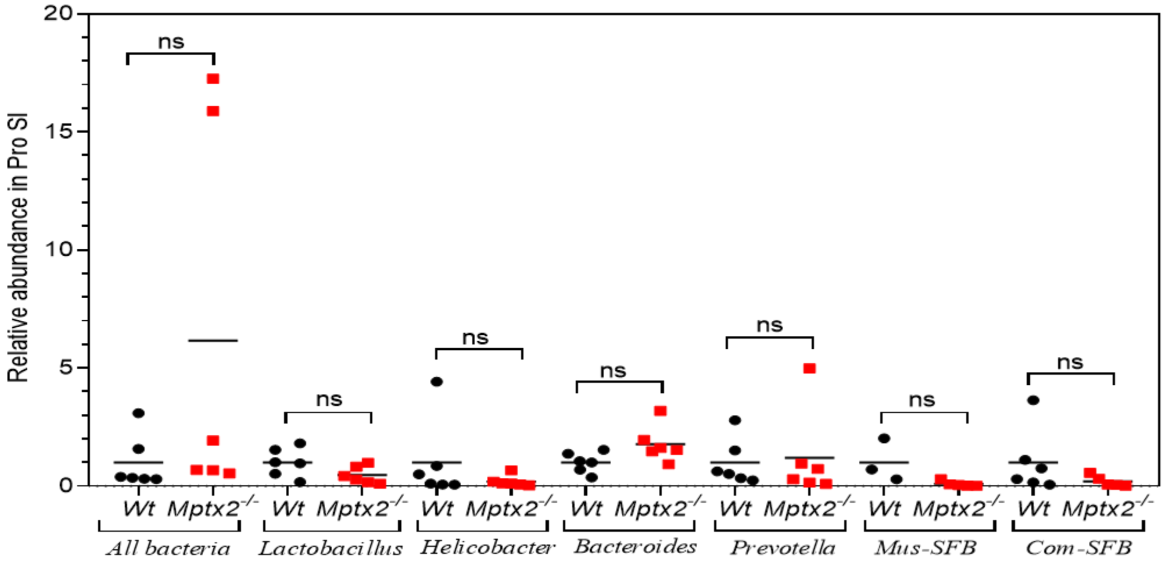
Relative abundance in Pro SI
20
15
10
5
0
ns
ns
ns
ns
ns
ns
ns
Wt Mptx2-/-
All bacteria
Lactobacillus
Helicobacter
Bacteroides
Prevotella
Mus-SFB
Com-SFB

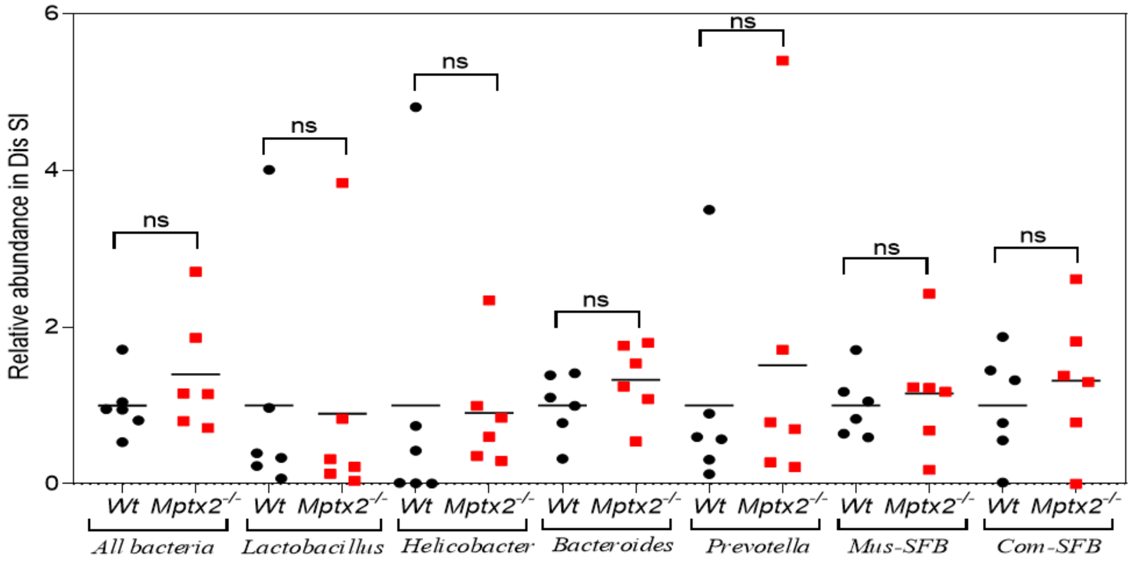
Relative abundance in Dis SI
6
4
2
0
ns
ns
ns
ns
ns
ns
ns
Wt Mptx2-/-
All bacteria
Lactobacillus
Helicobacter
Bacteroides
Prevotella
Mus-SFB
Com-SFB

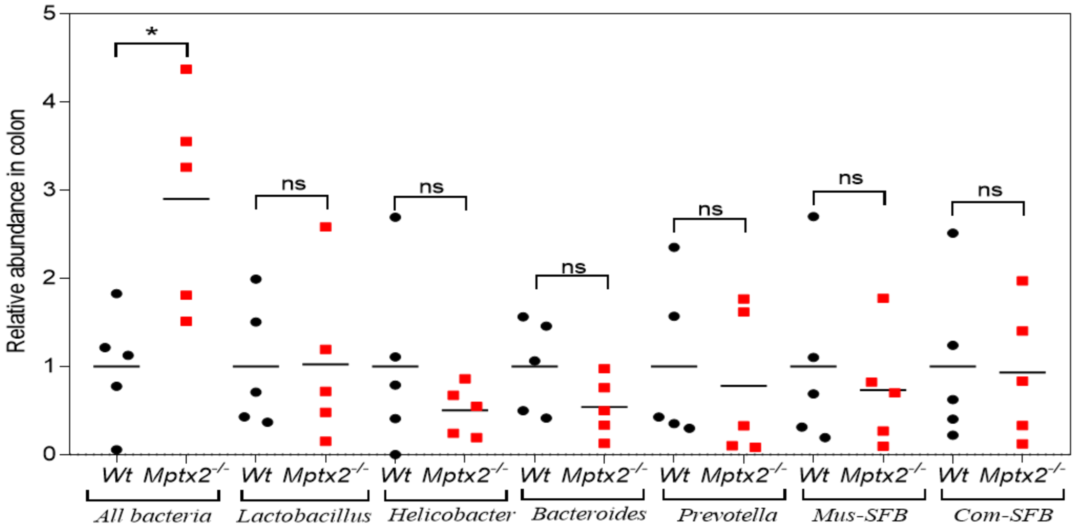
Relative abundance in colon
0
1
2
3
4
5
*
ns
Wt Mptx2-/-
All bacteria
Lactobacillus
Helicobacter
Bacteroides
Prevotella
Mus-SFB
Com-SFB

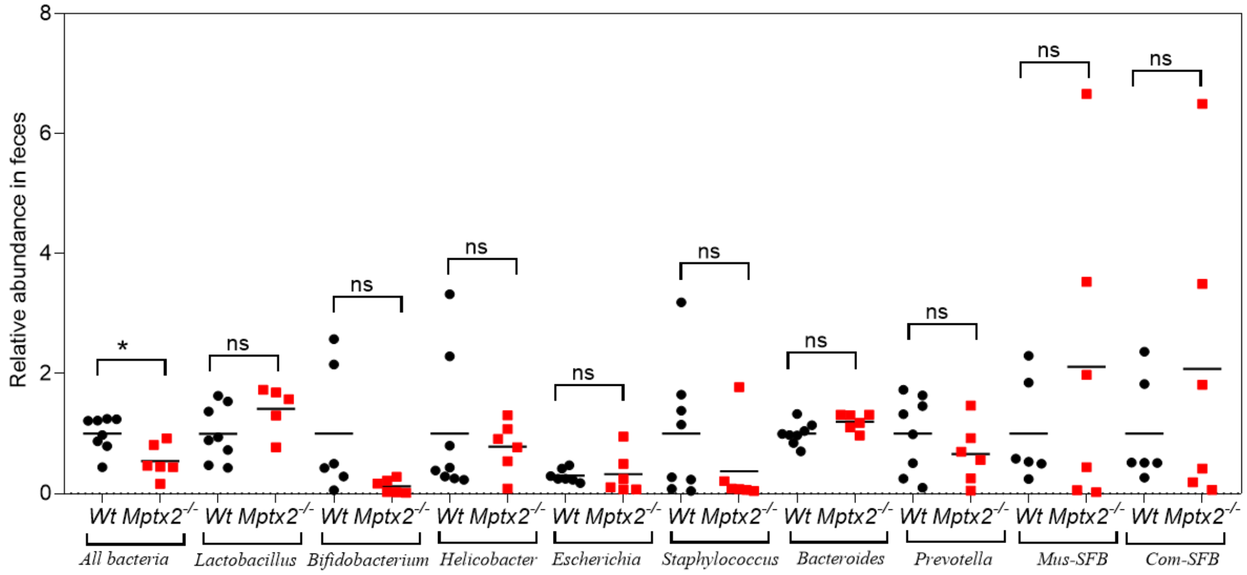
Relative abundance in feces
0
2
4
6
8
*
ns
Wt Mptx2-/-
All bacteria
Lactobacillus
Bifidobacterium
Helicobacter
Escherichia
Staphylococcus
Bacteroides
Prevotella
Mus-SFB
Com-SFB

Supplementary Figure 5. The *Mptx2*$^{-/-}$ mice had worsen intestinal injury after LPS-stimulation. **a** Quantitative real-time PCR (qRT-PCR) of *Mptx2* mRNA expression in the small intestine of mice (n = 10 - 15) subjected to LPS treatment. **b** Representative images of haematoxylin & eosin (H&E) staining on *Mptx2*$^{-/-}$ mice (n = 4) and *Wt* mice (n = 4) after LPS treatment. **c** The qualification of injury scores in panel (**b**). Unpaired two-tailed Student's *t* test with or without Welch's correction analysis for (**c**).ns, not significant, * p <0.05, ** p <0.01, *** p <0.001.

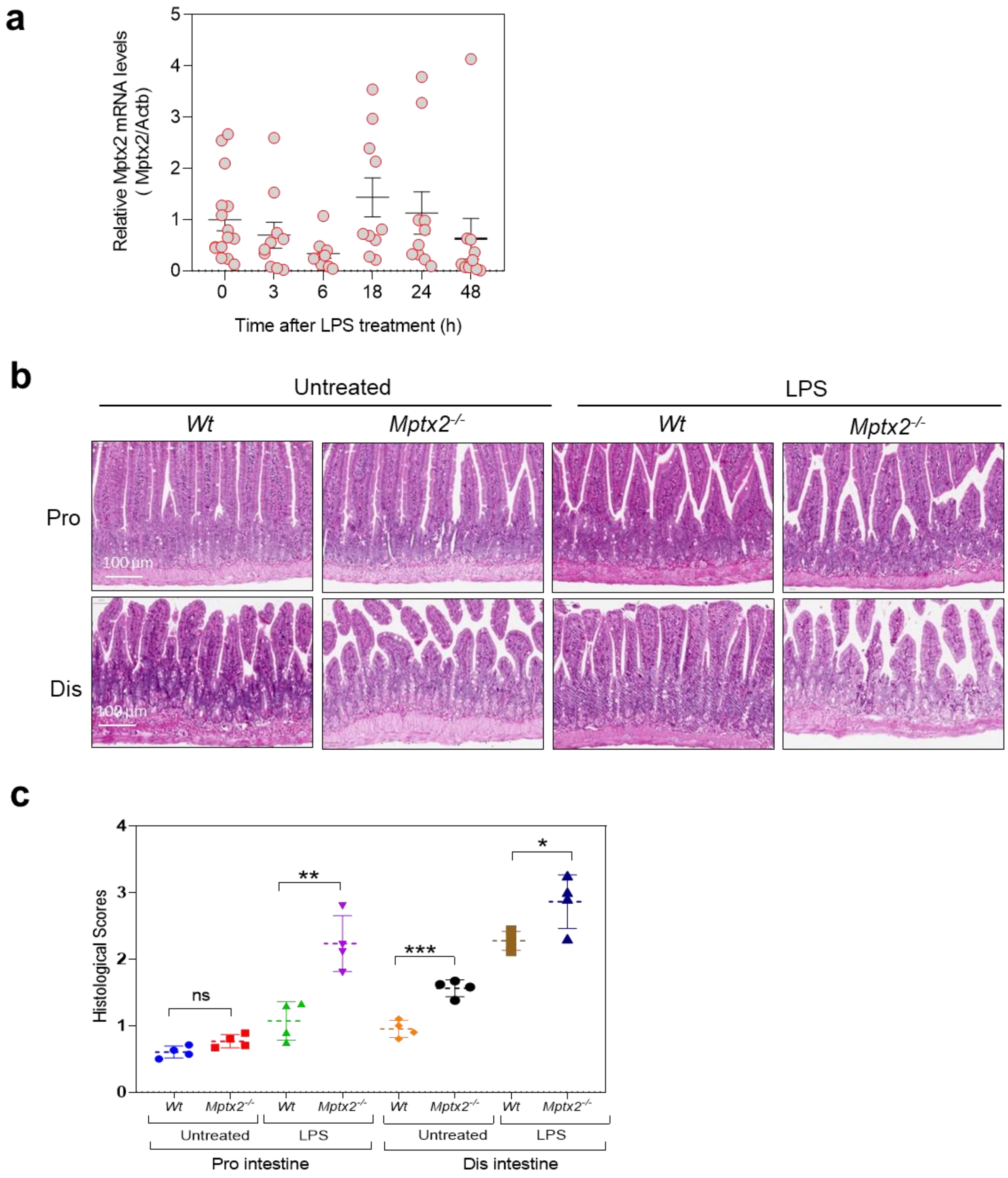

Supplementary Figure 6. The *Mptx2* deficiency decreased intestinal healing. a – b Representative images of Bromodeoxyuridine (BrdU) analysis for *Mptx2*$^{-/-}$ mice (n = 4) and *Wt* mice (n = 4) after LPS treatment. The qualification of BrdU-positive cells. **c** The *Lgr5* and Yap1 mRNA in the ileal mucosa of *Mptx2*$^{-/-}$ mice and *Wt* mice. Ordinary One-way ANOVA analysis for (**b**). Unpaired two-tailed Student's *t* test with Welch's correction analysis for (**c**). ns, not significant, * *p* <0.05.

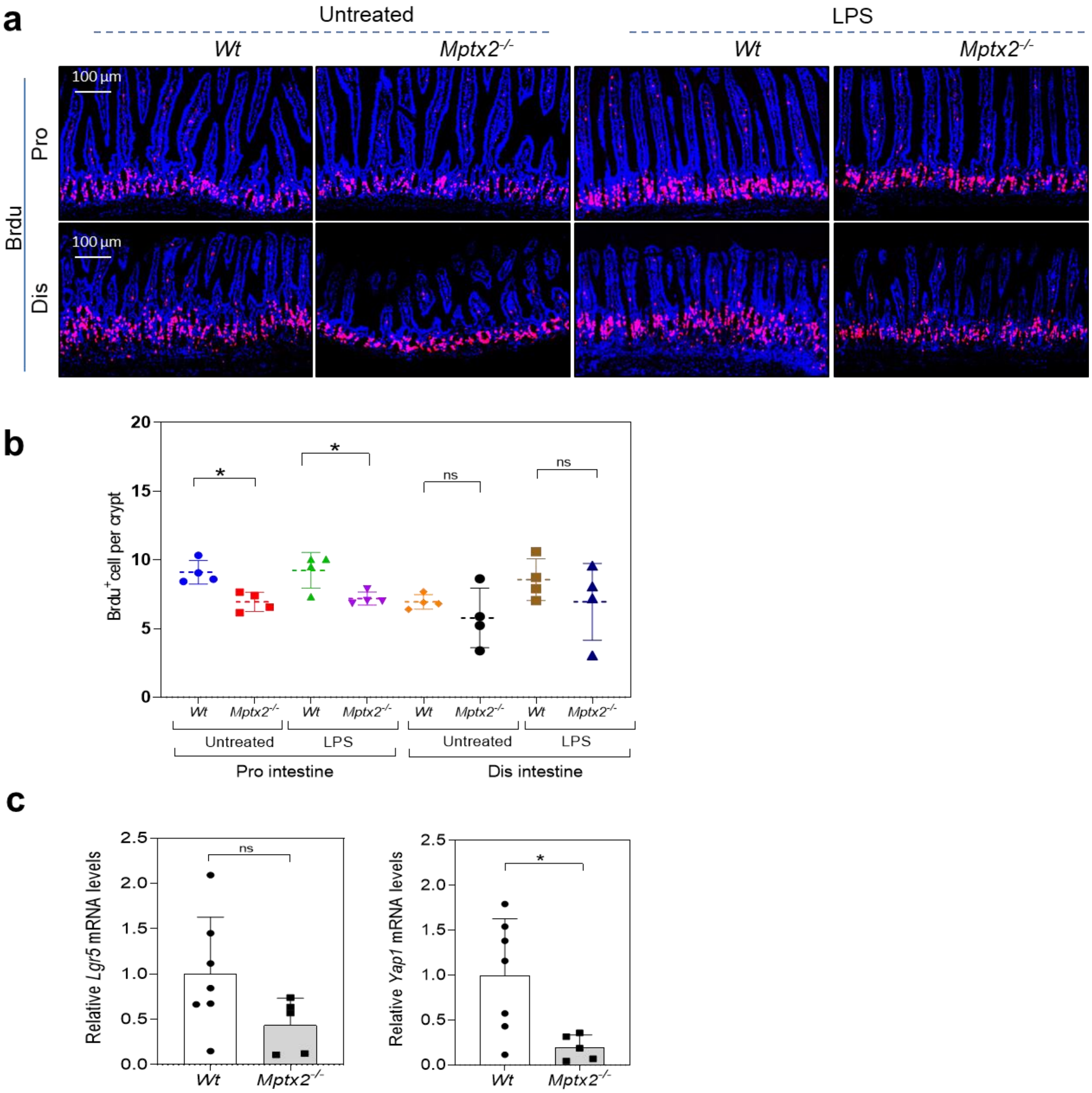

Supplementary Figure 7. The immunohistochemistry (IHC) of ZO-1 in small intestine of *Mptx2*$^{-/-}$ mice and *Wt* mice. a Representative images of ZO-1 immunohistochemistry (IHC) staining on dis small intestine of *Mptx2*$^{-/-}$ mice (n = 4) and *Wt* mice (n = 4) with or without LPS treatment. **b** The qualification of ZO-1 immunohistochemistry (IHC) staining in panel (**a**). Unpaired two-tailed Student's *t* test with or without Welch's correction analysis for (**b**).ns, not significant, * p <0.05

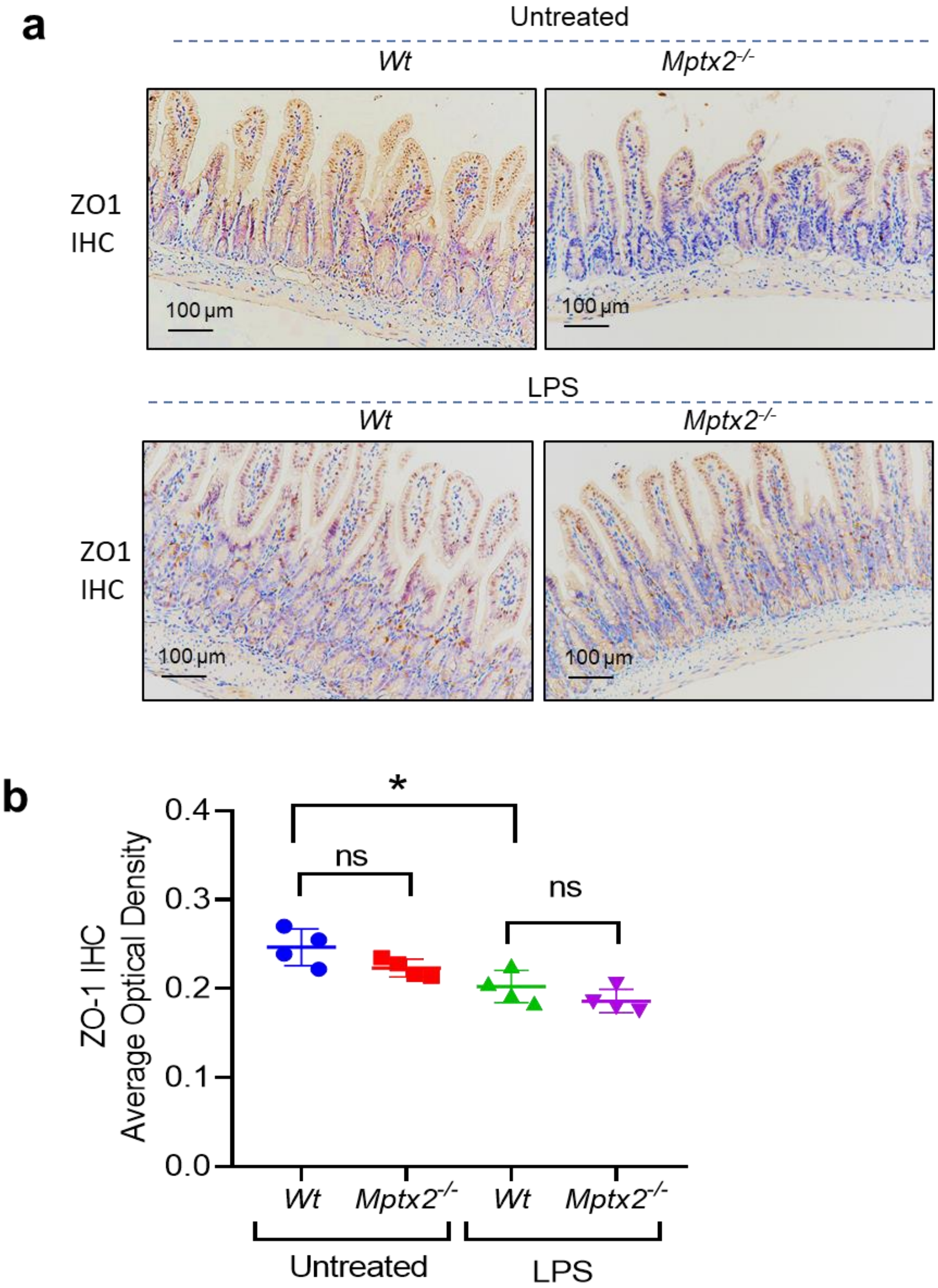

Supplementary Figure 8 Alteration of body weight and colon length in *Wt* and *Mptx2*$^{-/-}$ mice during the DSS-treatment. a Body weight changed during the giving the DSS. **b** Representative images of small intestine and colon from *Wt* and *Mptx2*$^{-/-}$ mice. **c** The changes of colon length from the *Wt* and *Mptx2*$^{-/-}$ mice. n =8 – 10. Ordinary One-way ANOVA analysis for (**c**). ns, not significant, * $p < 0.05$, ** $p < 0.01$, *** $p < 0.001$.

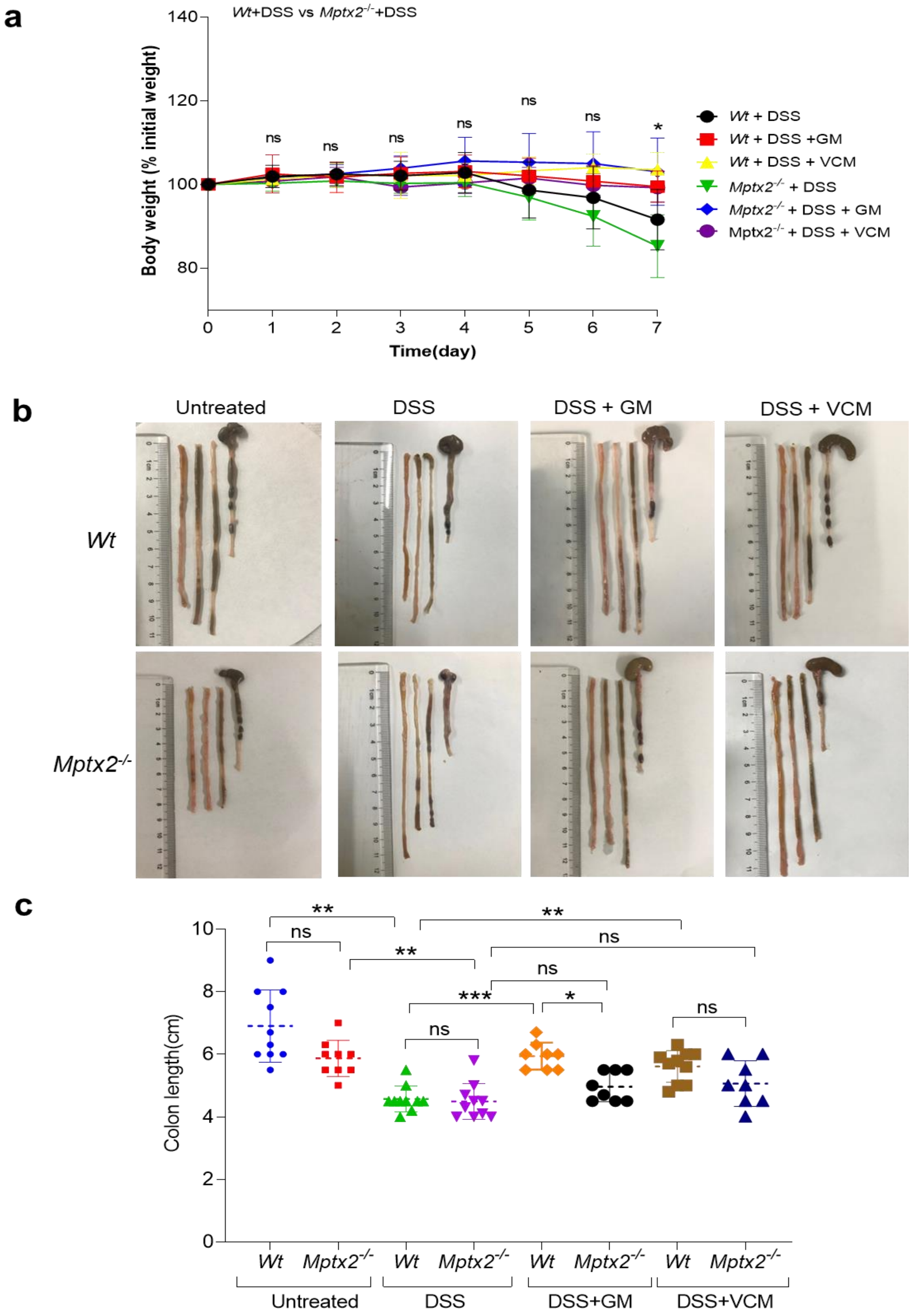
a
Wt+DSS vs Mptx2-/-+DSS
Body weight (% initial weight)
Time(day)
ns
*
Wt + DSS
Wt + DSS +GM
Wt + DSS + VCM
Mptx2-/- + DSS
Mptx2-/- + DSS + GM
Mptx2-/- + DSS + VCM
b
Untreated
DSS
DSS + GM
DSS + VCM
Wt
Mptx2-/-
c
Colon length(cm)
**
ns
***
*
Wt
Mptx2-/-
Untreated
DSS
DSS+GM
DSS+VCM

Supplementary Figure 9 *Mptx2* loss worsens DSS-induced colon inflammation. qRT-PCR analysis of inflammatory genes mRNA expression in colonic mucosa from both *Mptx2*$^{-/-}$ mice and *Wt* mice (each group, n = 4 - 8). Unpaired two-tailed Student's *t* test with Welch's correction analysis for Supplementary Figure 9. ns, not significant, * *p* <0.05 ,** *p* <0.01, *** *p* <0.001, **** *p* <0.0001.

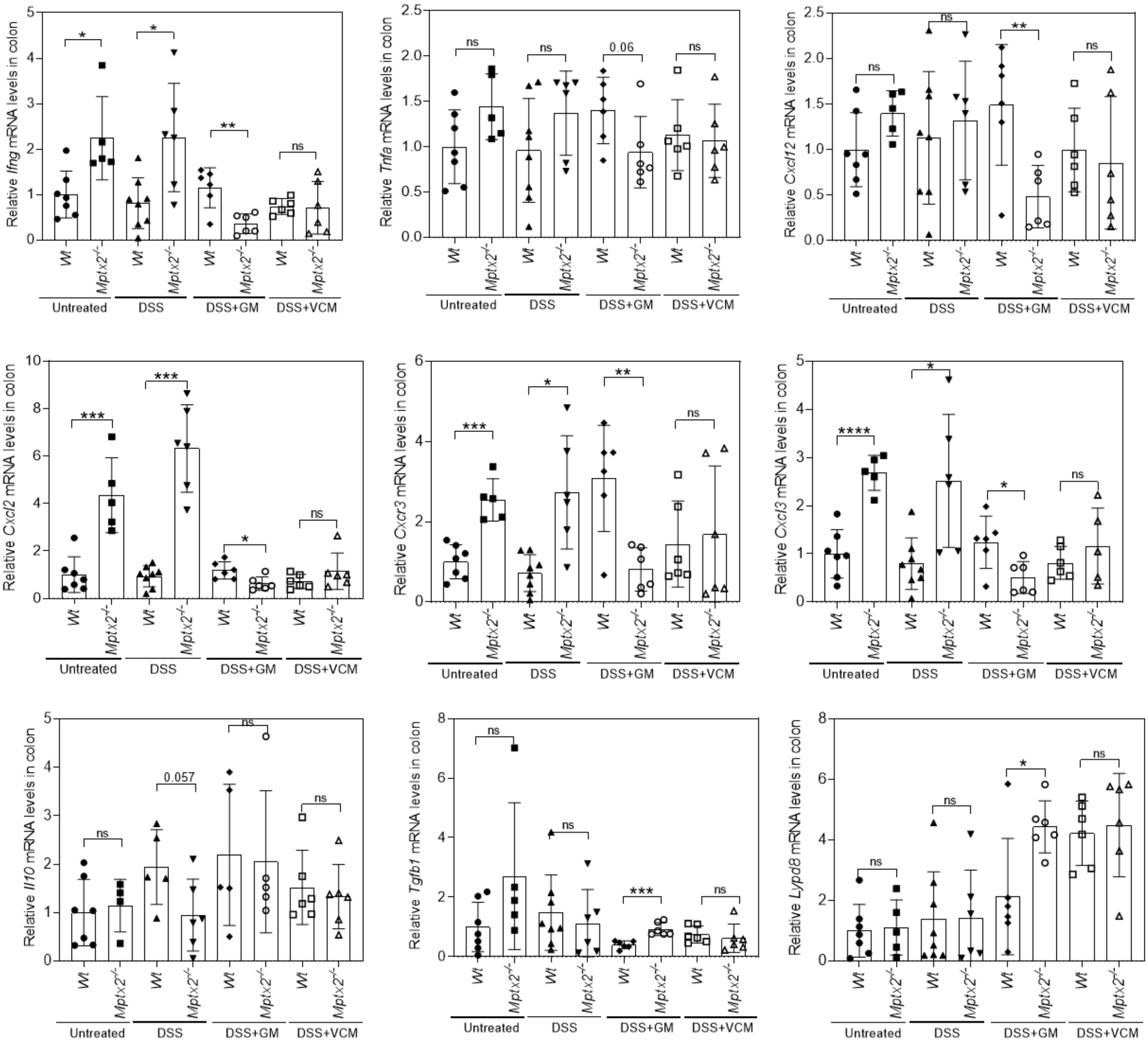

Supplementary Figure 10 Goblet cells changed between *Wt* and *Mptx2*$^{-/-}$ mice. a Representative images of Alcian blue/periodic acid Schiff base (AB-PAS) staining on colonic Swiss-roll. **b** The quantification of Panel A (n = 6 - 10).Ordinary One-way ANOVA analysis for (**b**). ns, not significant, * p <0.05, *** p <0.001, **** p <0.0001.

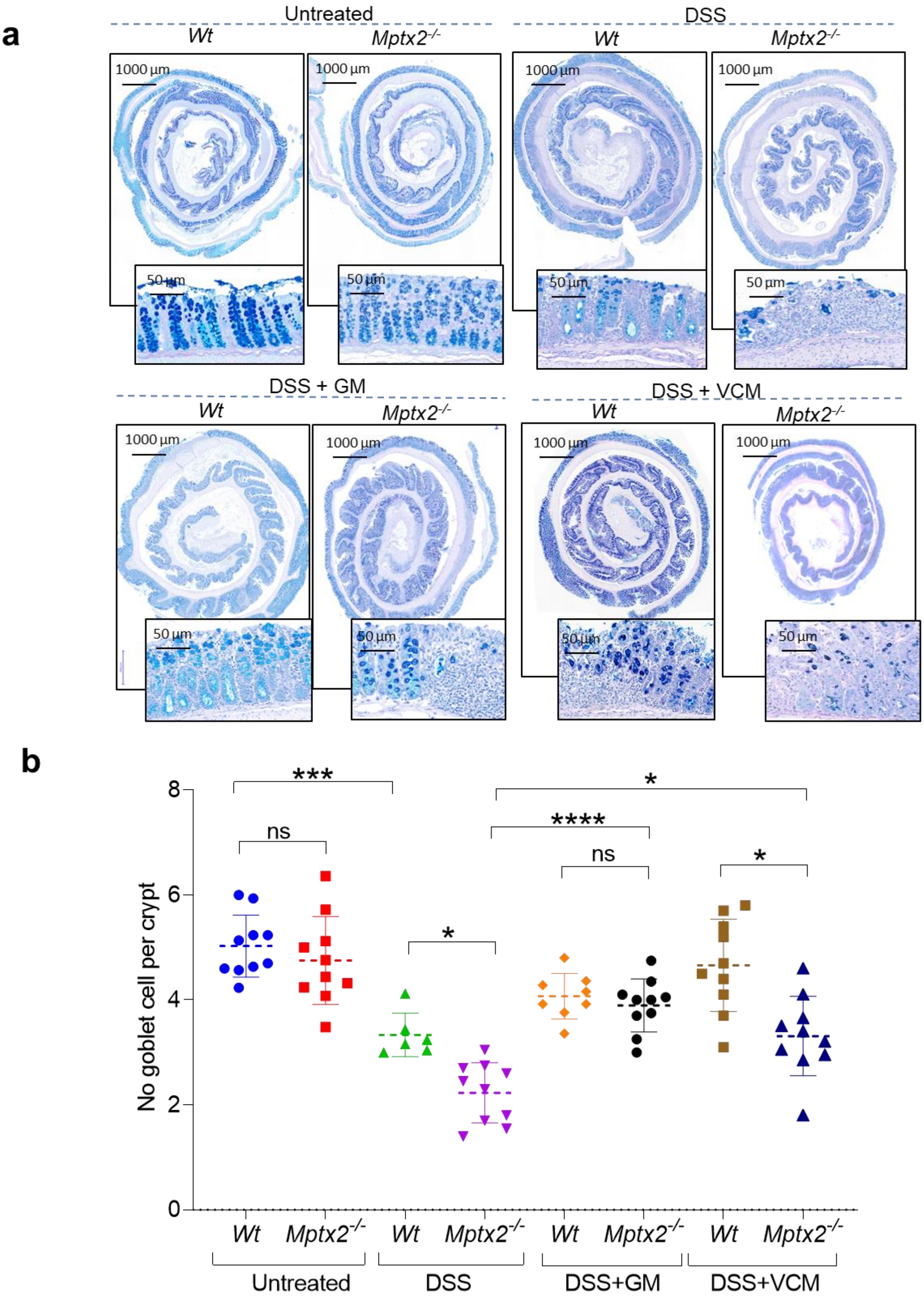

3. Supplementary Figure 11 Original WB bands in Figures and gel blots

Figure 1b

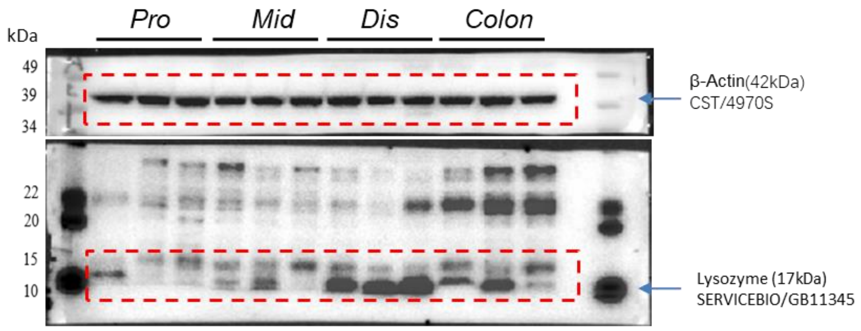

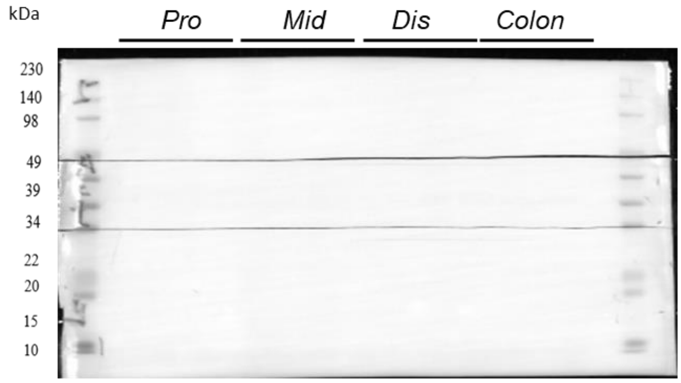

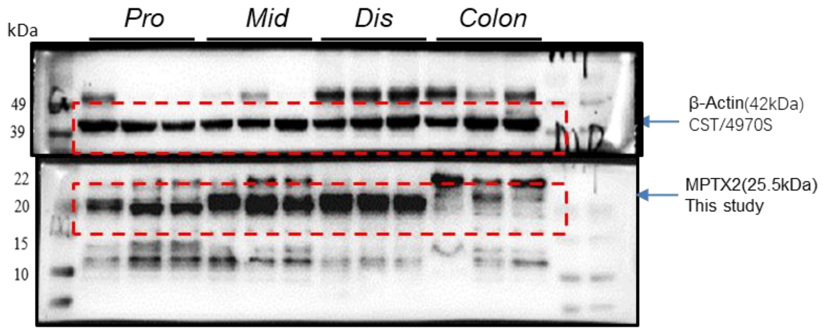

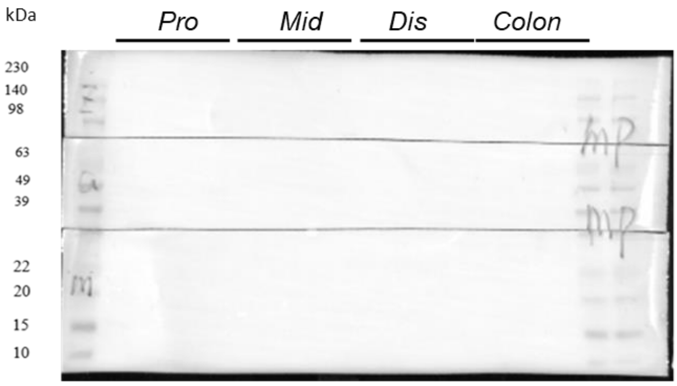

Figure 6d

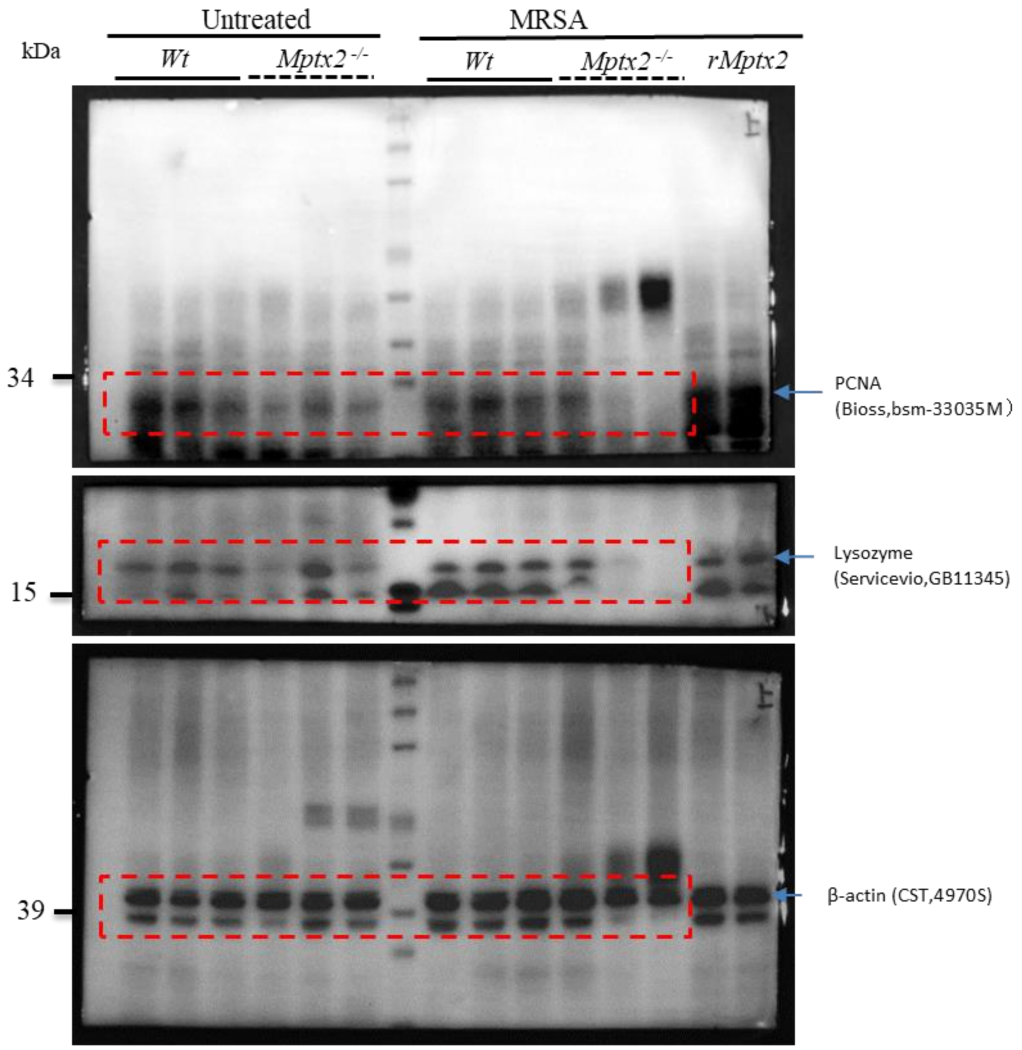

Untreated
MRSA
kDa
Wt
Mptx2 -/-
Wt
Mptx2 -/-
rMptx2
34
15
39
PCNA
(Bioss,bsm-33035M）
Lysozyme
(Servicevio,GB11345)
β-actin (CST,4970S)

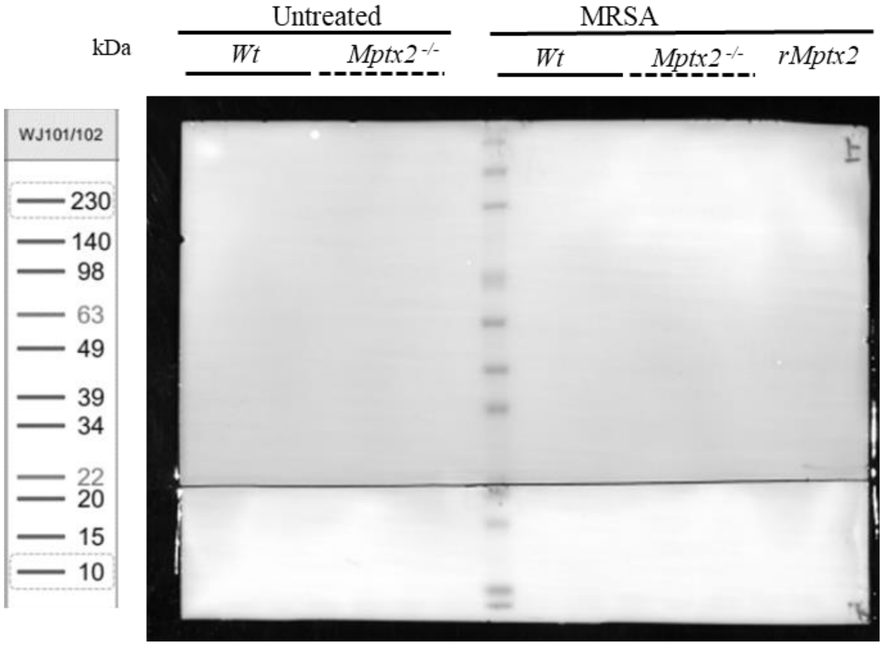

Untreated
MRSA
kDa
Wt
Mptx2 -/-
Wt
Mptx2 -/-
rMptx2
WJ101/102
230
140
98
63
49
39
34
22
20
15
10

Figure 6h

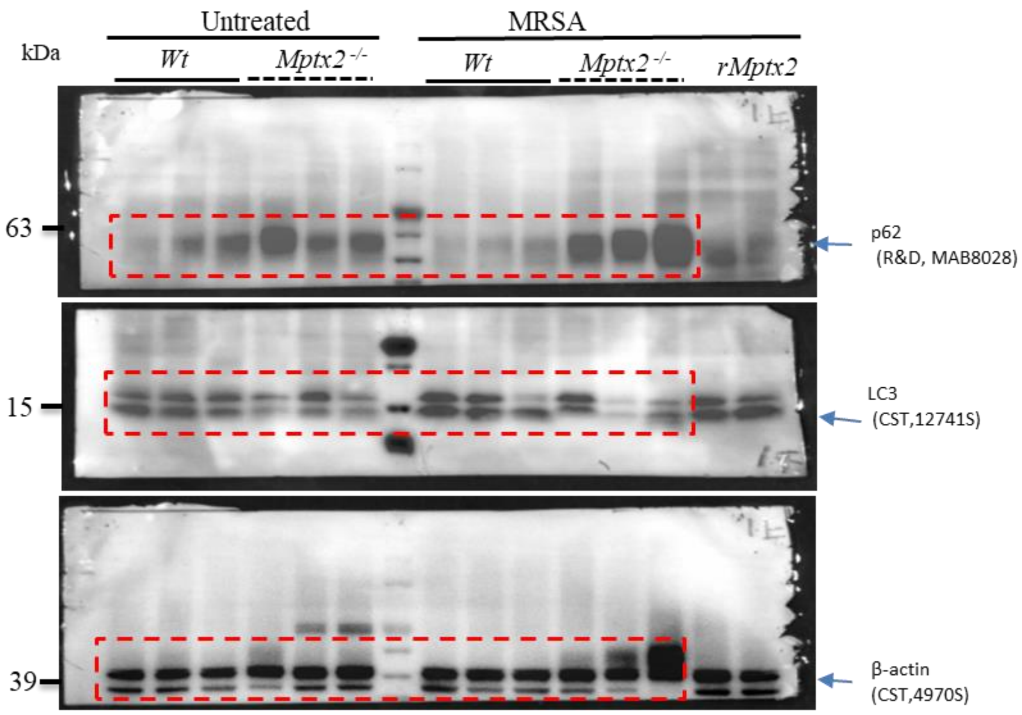
Untreated
MRSA
kDa
Wt
Mptx2 -/-
Wt
Mptx2 -/-
rMptx2
63
15
39
p62
(R&D, MAB8028)
LC3
(CST,12741S)
β-actin
(CST,4970S)

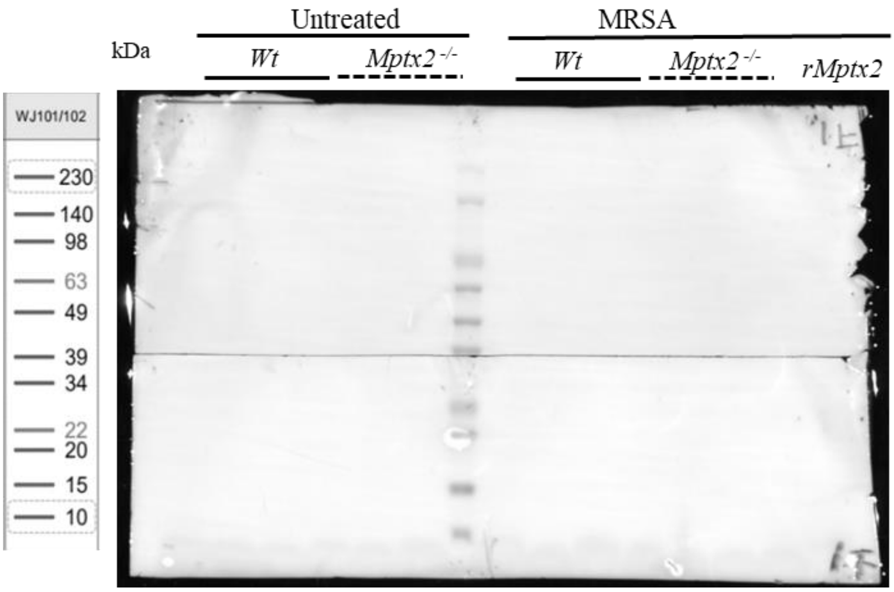
Untreated
MRSA
kDa
Wt
Mptx2 -/-
Wt
Mptx2 -/-
rMptx2
WJ101/102
230
140
98
63
49
39
34
22
20
15
10

Figure 7a

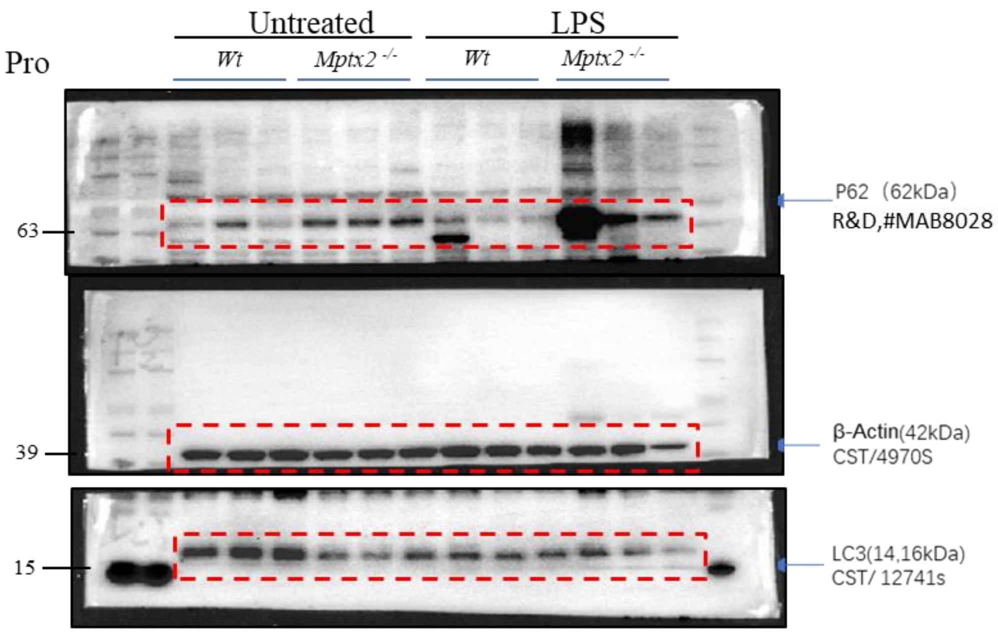
Untreated
LPS
Pro
Wt
Mptx2 -/-
Wt
Mptx2 -/-
63
39
15
P62（62kDa)
R&D,#MAB8028
β-Actin(42kDa)
CST/4970S
LC3(14,16kDa)
CST/ 12741s

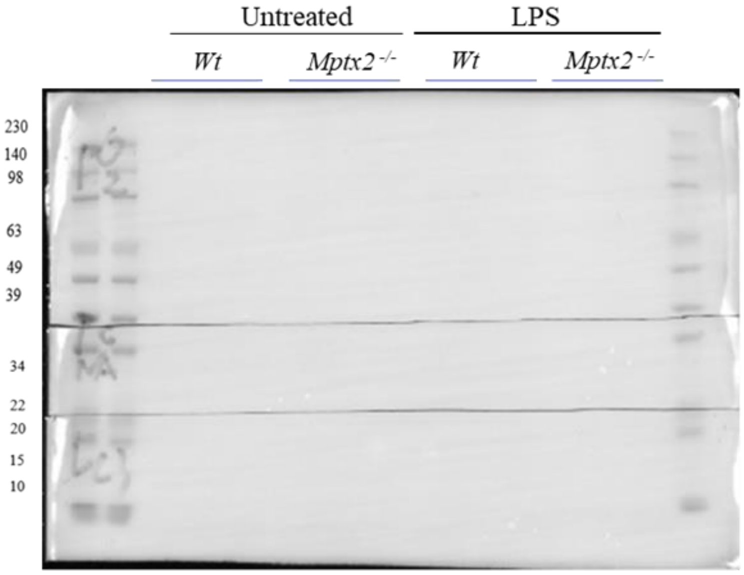
Untreated
LPS
Wt
Mptx2 -/-
Wt
Mptx2 -/-
230
140
98
63
49
39
34
22
20
15
10

Figure 7a

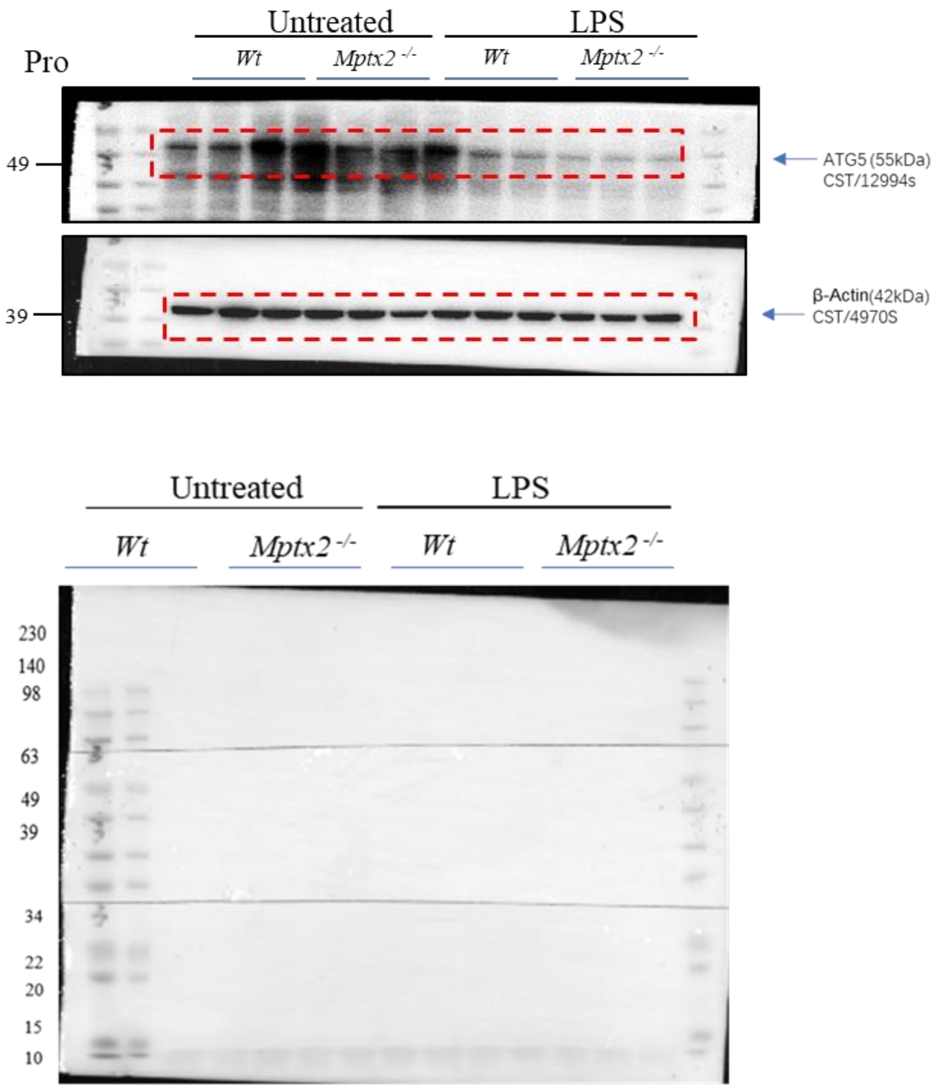

Untreated
LPS
Wt
Mptx2 -/-
Wt
Mptx2 -/-
Pro
49
39
ATG5 (55kDa)
CST/12994s
β-Actin(42kDa)
CST/4970S
Untreated
LPS
Wt
Mptx2 -/-
Wt
Mptx2 -/-
230
140
98
63
49
39
34
22
20
15
10

Figure 7a

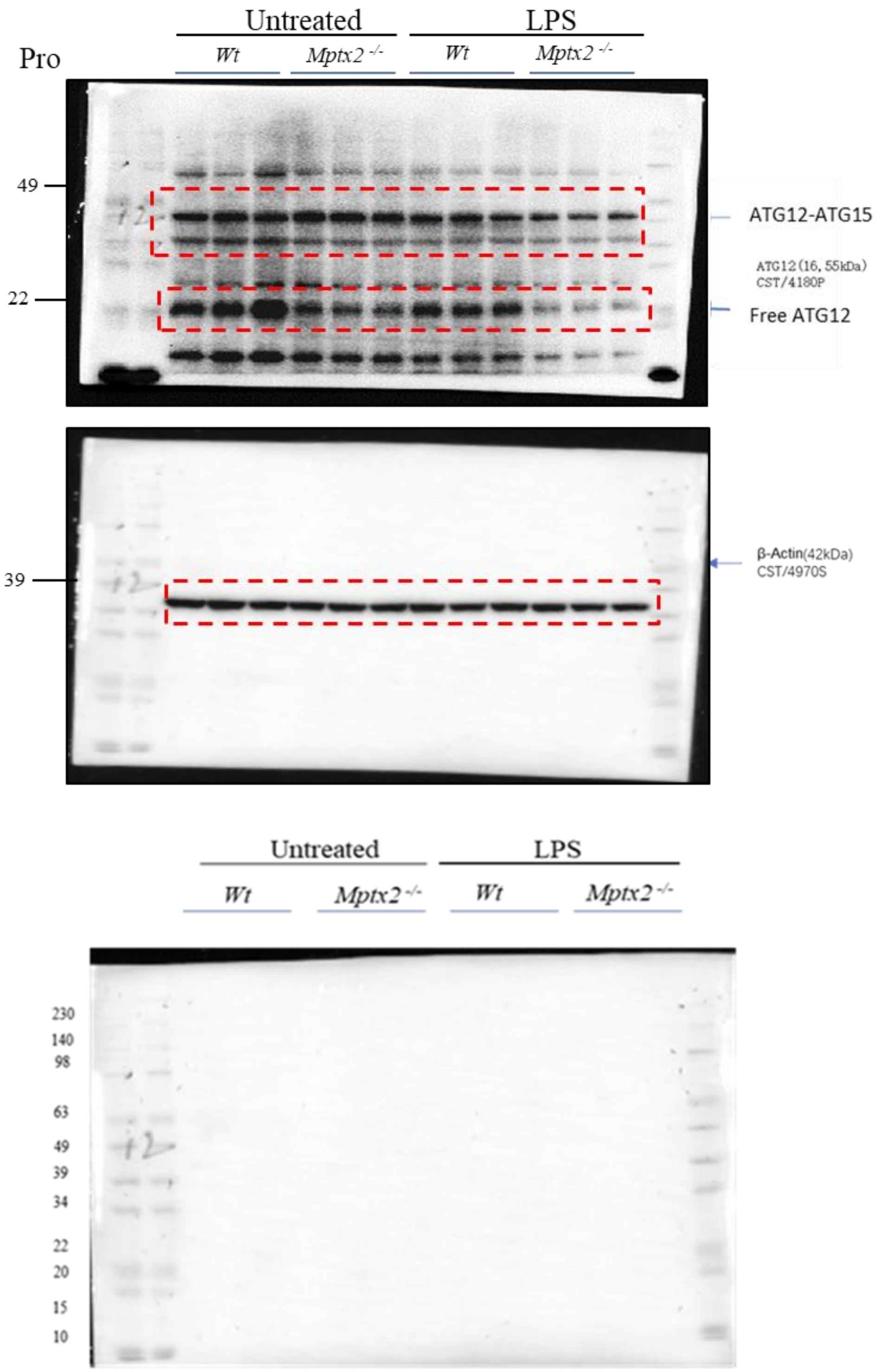

Untreated
LPS
Pro
Wt
Mptx2 -/-
Wt
Mptx2 -/-
49
22
ATG12-ATG15
ATG12(16, 55kDa)
CST/4180P
Free ATG12
39
β-Actin(42kDa)
CST/4970S
Untreated
LPS
Wt
Mptx2 -/-
Wt
Mptx2 -/-
230
140
98
63
49
39
34
22
20
15
10

Figure 7a

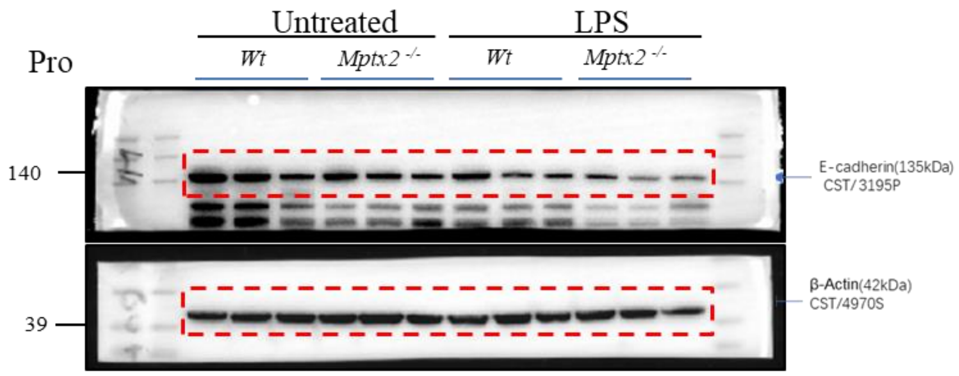

Untreated
LPS
Wt
Mptx2 -/-
Wt
Mptx2 -/-
Pro
140
39
E-cadherin(135kDa)
CST/3195P
β-Actin(42kDa)
CST/4970S

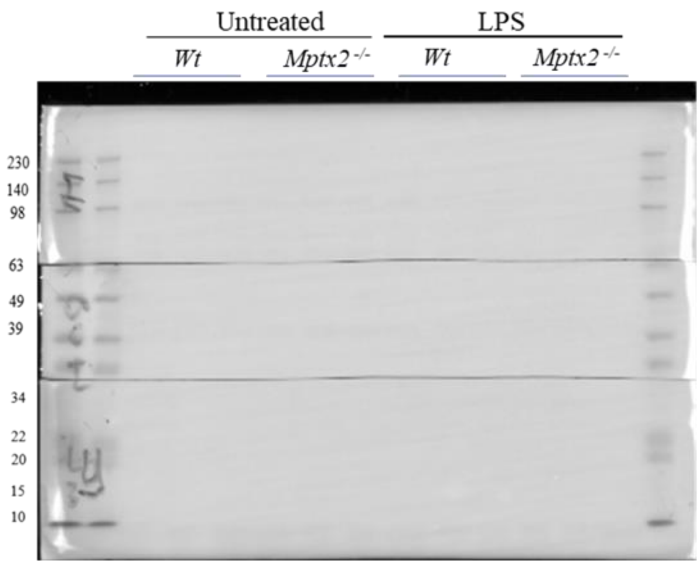

Untreated
LPS
Wt
Mptx2 -/-
Wt
Mptx2 -/-
230
140
98
63
49
39
34
22
20
15
10

Figure 7a

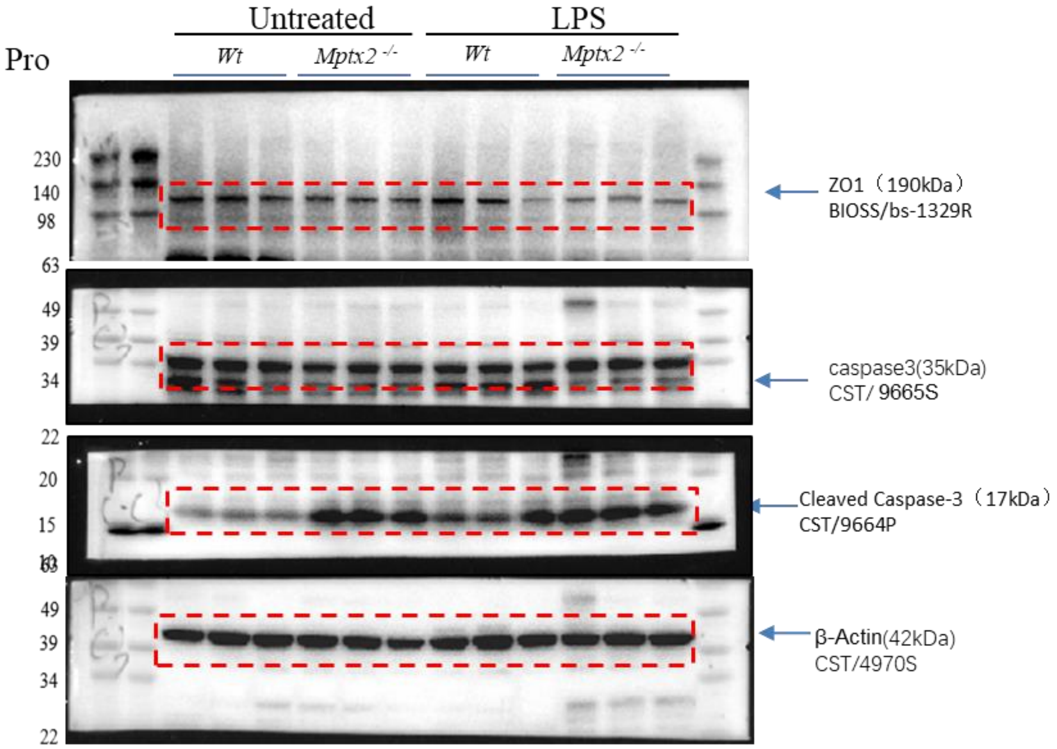
Untreated
LPS
Wt
Mptx2 -/-
Wt
Mptx2 -/-
Pro
230
140
98
63
49
39
34
22
20
15
10
63
49
39
34
22
ZO1（190kDa）
BIOSS/bs-1329R
caspase3(35kDa)
CST/ 9665S
Cleaved Caspase-3（17kDa）
CST/9664P
β-Actin(42kDa)
CST/4970S

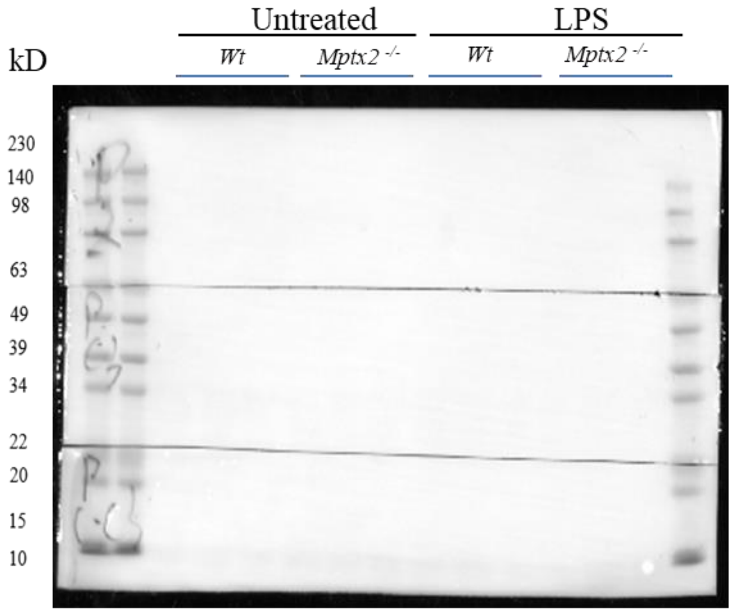
Untreated
LPS
Wt
Mptx2 -/-
Wt
Mptx2 -/-
kD
230
140
98
63
49
39
34
22
20
15
10

Figure 7a

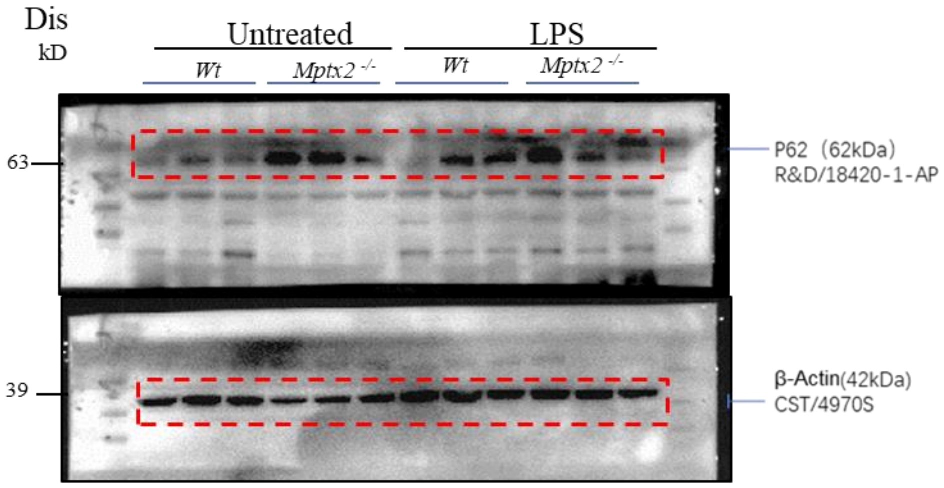
Dis
kD
Untreated
LPS
Wt
Mptx2 -/-
Wt
Mptx2 -/-
63
39
P62（62kDa）
R&D/18420-1-AP
β-Actin(42kDa)
CST/4970S

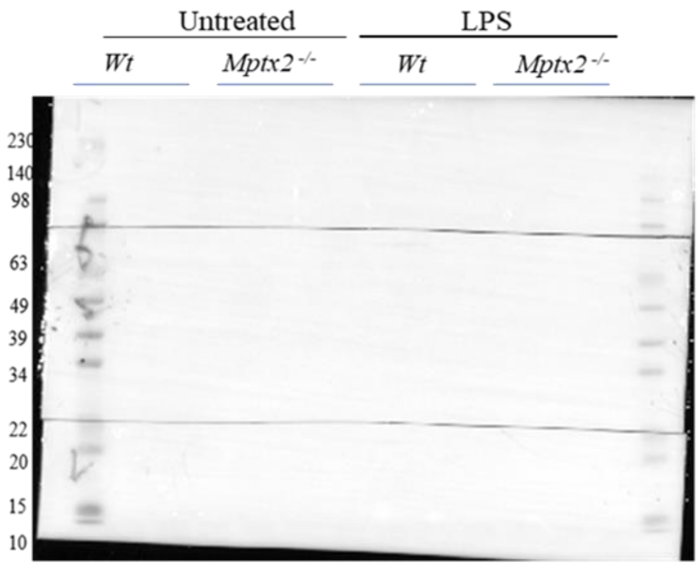
Untreated
LPS
Wt
Mptx2 -/-
Wt
Mptx2 -/-
140
98
63
49
39
34
22
20
15
10

Figure 7a

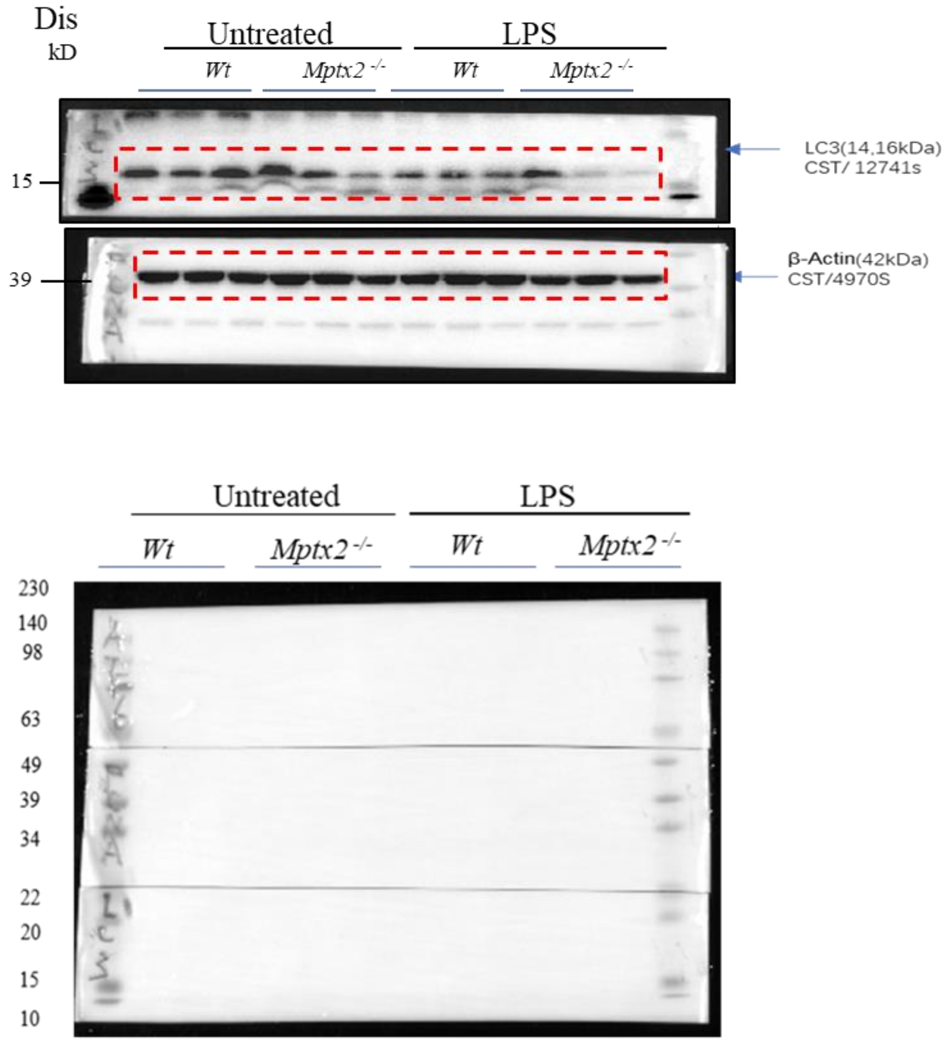
Dis
kD
Untreated
LPS
Wt
Mptx2 -/-
Wt
Mptx2 -/-
15
39
LC3(14,16kDa)
CST/ 12741s
β-Actin(42kDa)
CST/4970S
Untreated
LPS
Wt
Mptx2 -/-
Wt
Mptx2 -/-
230
140
98
63
49
39
34
22
20
15
10

Figure 7a

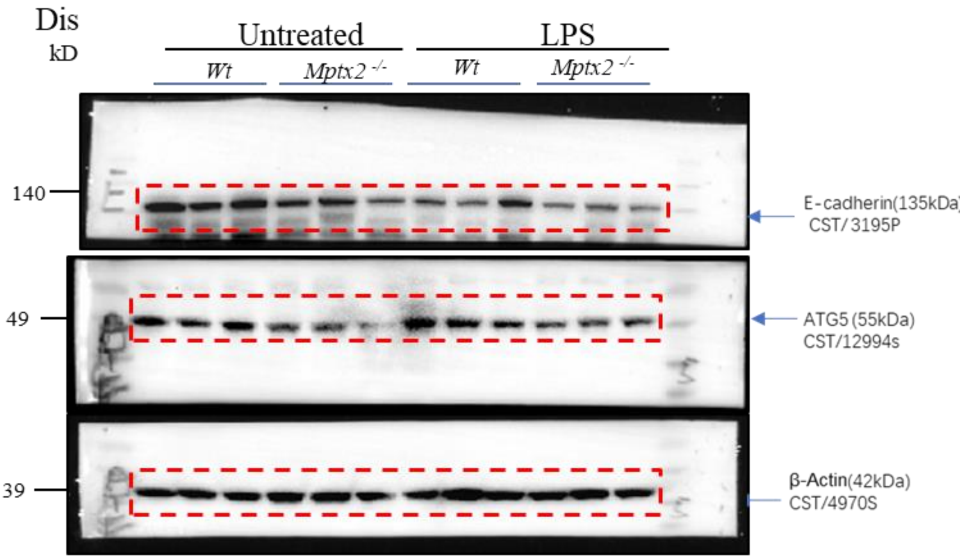

Dis
kD
Untreated
LPS
Wt
Mptx2 -/-
Wt
Mptx2 -/-
140
49
39
E-cadherin(135kDa)
CST/3195P
ATG5 (55kDa)
CST/12994s
β-Actin(42kDa)
CST/4970S

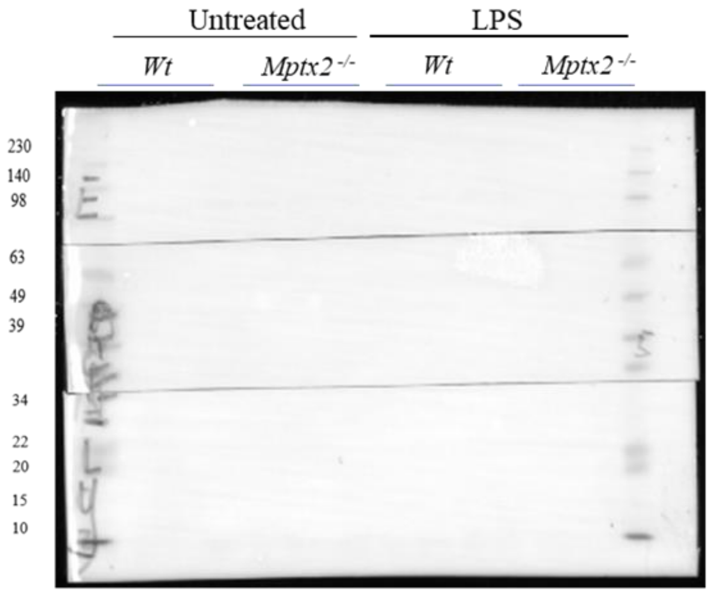

Untreated
LPS
Wt
Mptx2 -/-
Wt
Mptx2 -/-
230
140
98
63
49
39
34
22
20
15
10

Figure 7a

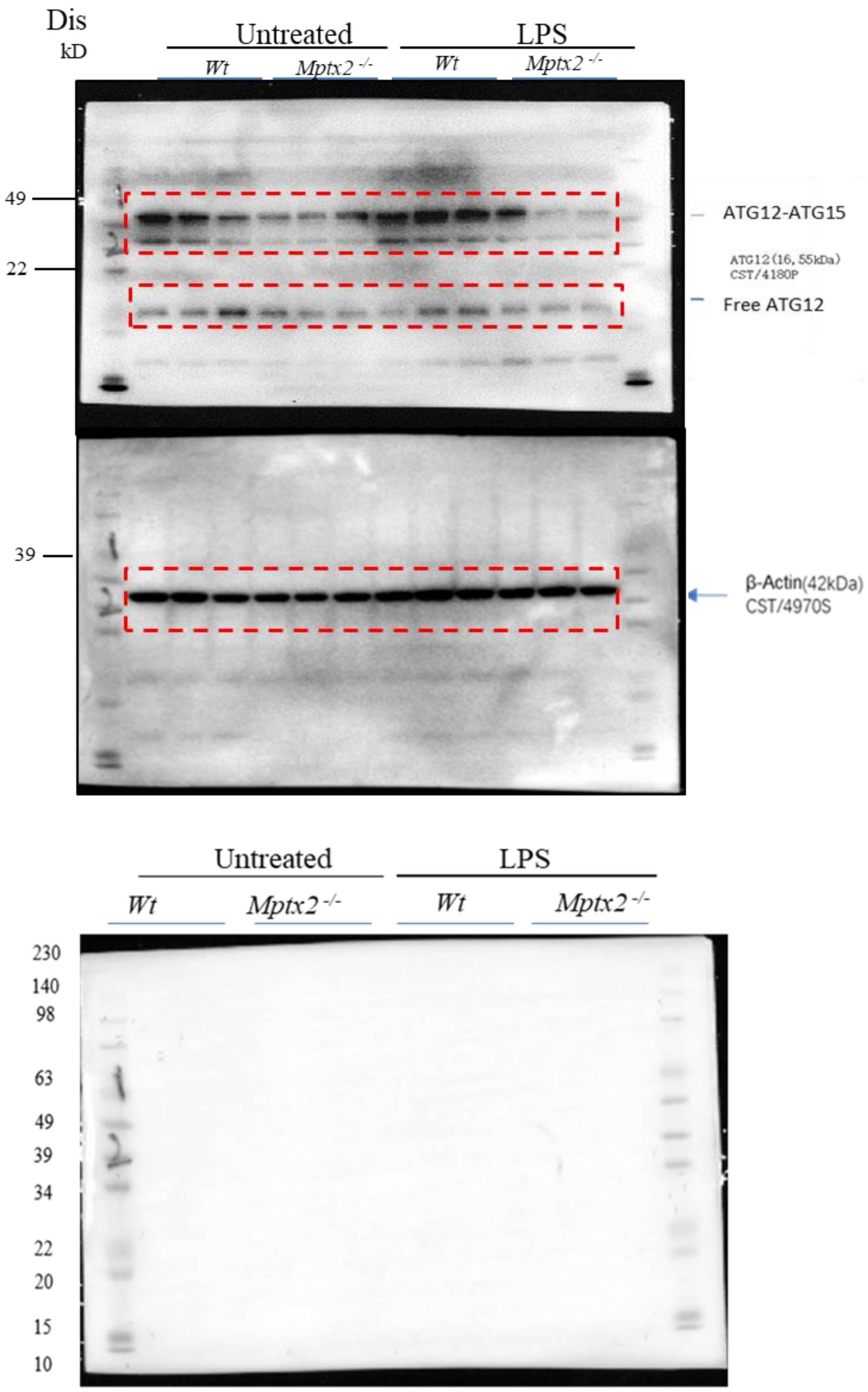
Dis
kD
Untreated
LPS
Wt
Mptx2 -/-
Wt
Mptx2 -/-
49
22
ATG12-ATG15
ATG12 (16, 55kDa)
CST/4180P
Free ATG12
39
β-Actin(42kDa)
CST/4970S
Untreated
LPS
Wt
Mptx2 -/-
Wt
Mptx2 -/-
230
140
98
63
49
39
34
22
20
15
10

Figure 7a

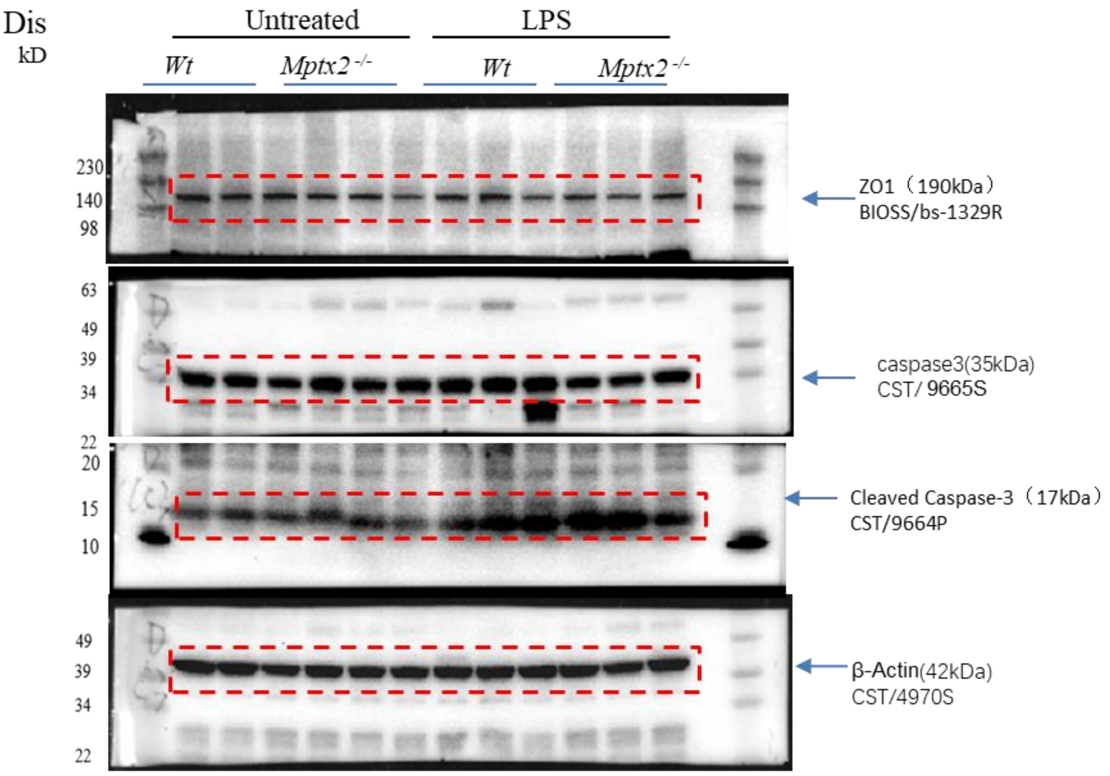

Dis
kD
Untreated
LPS
Wt
Mptx2 -/-
Wt
Mptx2 -/-
230
140
98
ZO1（190kDa）
BIOSS/bs-1329R
63
49
39
34
caspase3(35kDa)
CST/ 9665S
22
20
15
10
Cleaved Caspase-3（17kDa）
CST/9664P
49
39
34
22
β-Actin(42kDa)
CST/4970S

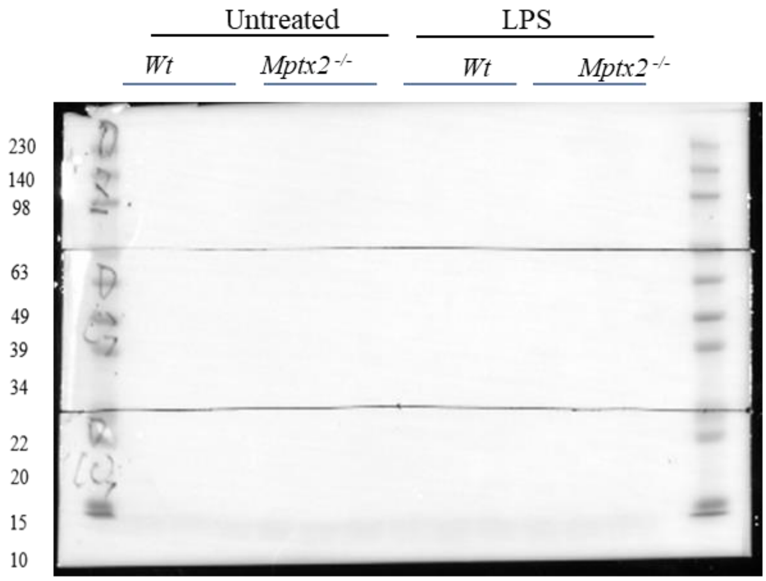

Untreated
LPS
Wt
Mptx2 -/-
Wt
Mptx2 -/-
230
140
98
63
49
39
34
22
20
15
10

Supplementary Figure 1b

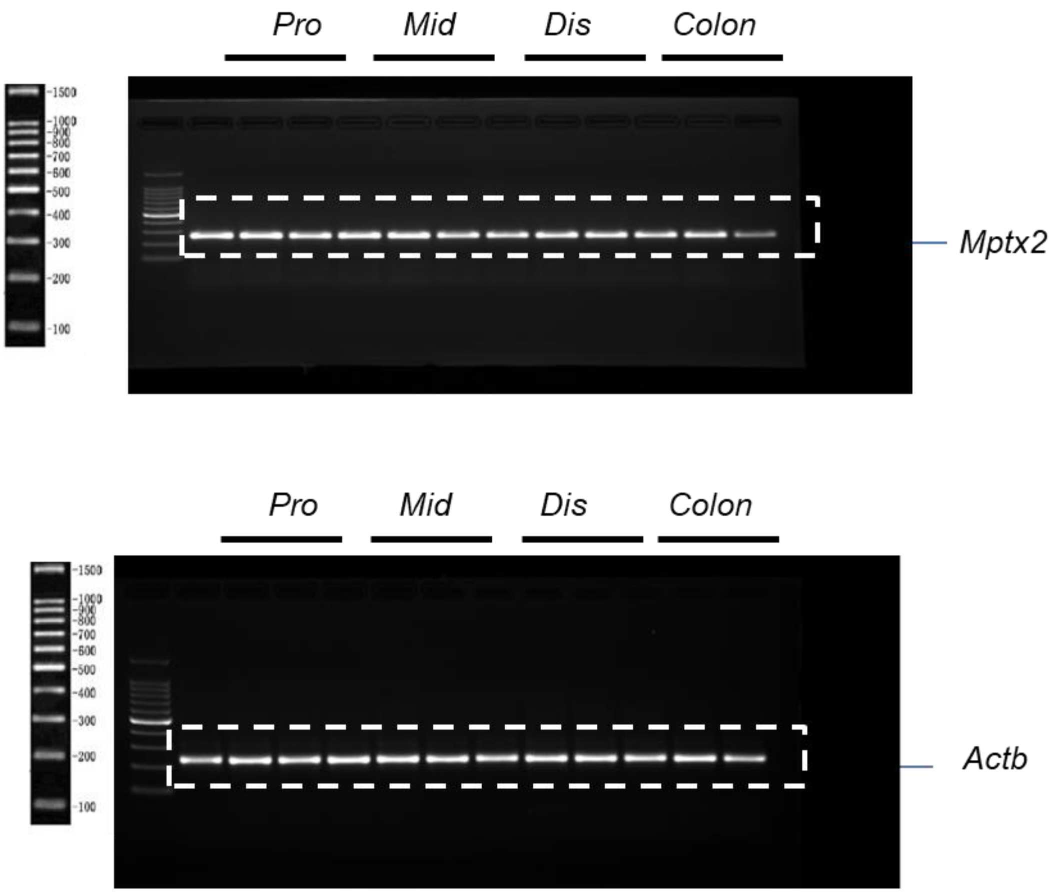

Supplementary Figure 1c

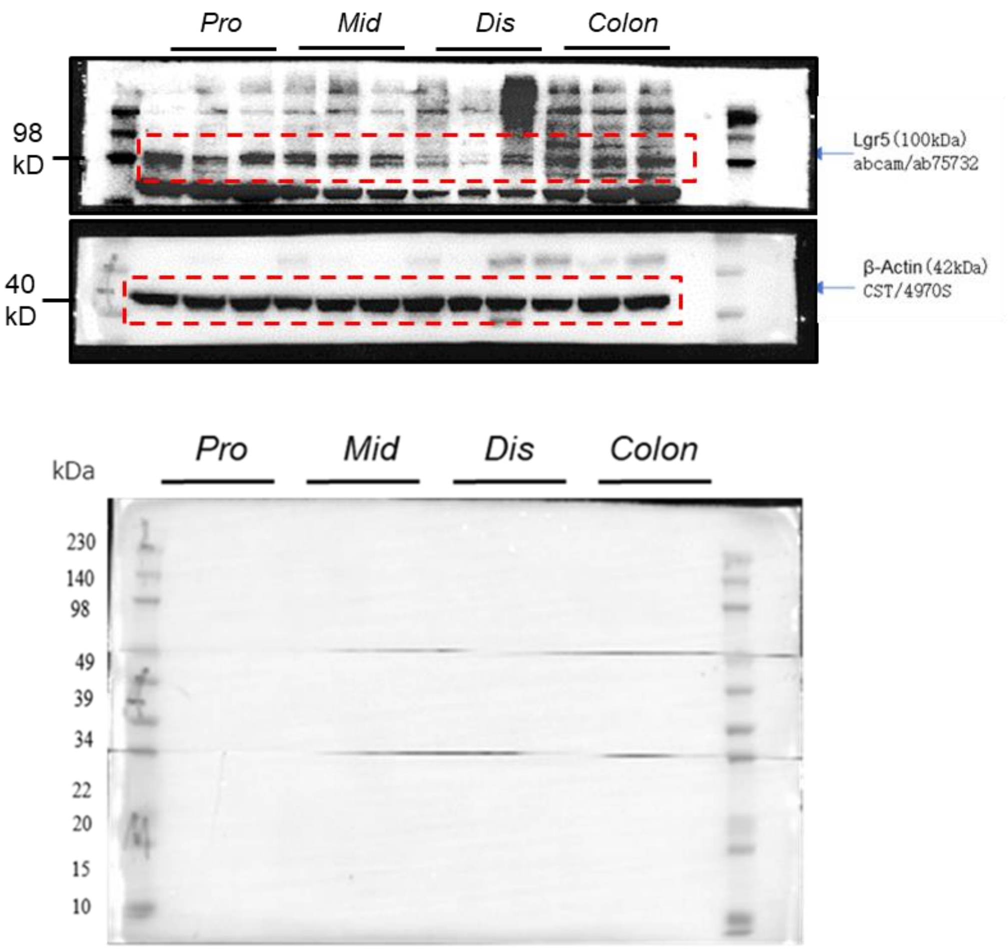

Supplementary Figure 2a

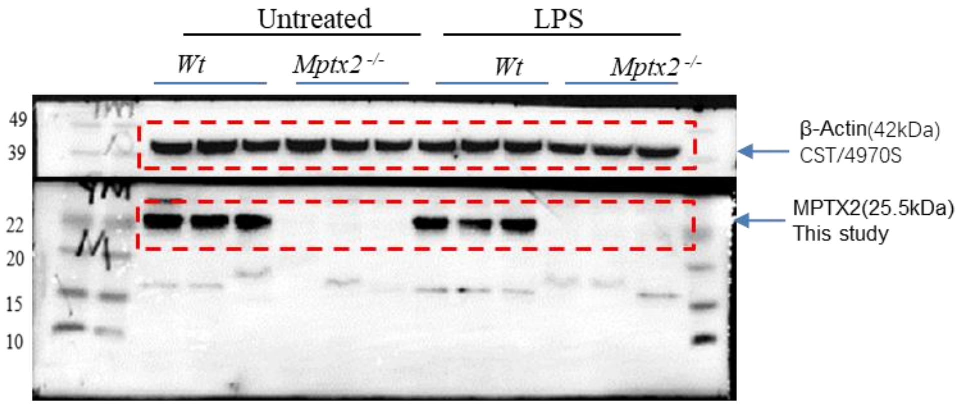

Dis small intestine

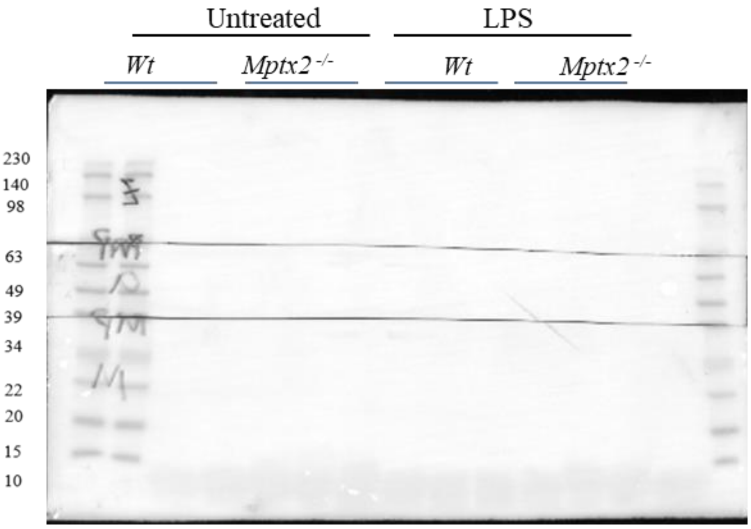

Supplement: Supplementary file 1 — Supplementary Information [file 42003_2024_5785_MOESM1_ESM.pdf]
