# Supplementary material for: Loss of Mptx2 alters bacteria composition and intestinal homeostasis potentially by impairing autophagy
Source: Commun Biol. 2024 Jan 13;7:94. doi: 10.1038/s42003-024-05785-7 (PMC10787791; doi:10.1038/s42003-024-05785-7)
Supplement: Supplementary file 2 — Description of Additional Supplementary Files [file 42003_2024_5785_MOESM2_ESM.pdf]

Description of Additional Supplementary Files

File name: Supplementary Data 1
Description: Source data and statistical analysis for all graphs.
